# NRF2 promotes radiation resistance by cooperating with TOPBP1 to activate the ATR-CHK1 signaling pathway

**DOI:** 10.7150/thno.88899

**Published:** 2024-01-01

**Authors:** Xiaohui Sun, Mingxin Dong, Jiale Li, Yuxiao Sun, Yu Gao, Yan Wang, Liqing Du, Yang Liu, Kaihua Ji, Ningning He, Jinhan Wang, Manman Zhang, Huijuan Song, Chang Xu, Qiang Liu

**Affiliations:** Tianjin Key Laboratory of Radiation Medicine and Molecular Nuclear Medicine, Institute of Radiation Medicine, Chinese Academy of Medical Sciences and Peking Union Medical College, Tianjin 300192, China.

**Keywords:** Ionizing radiation, NRF2, NSCLC, ATR, RPA32, TOPBP1

## Abstract

**Background:** Radiation resistance is the main limitation of the application of radiotherapy. Ionizing radiation (IR) kills cancer cells mainly by causing DNA damage, particularly double-strand breaks (DSBs). Radioresistant cancer cells have developed the robust capability of DNA damage repair to survive IR. Nuclear factor erythroid 2-related factor 2 (NRF2) has been correlated with radiation resistance. We previously reported a novel function of NRF2 as an ATR activator in response to DSBs. However, little is known about the mechanism that how NRF2 regulates DNA damage repair and radiation resistance.

**Methods:** The TCGA database and tissue microarray were used to analyze the correlation between NRF2 and the prognosis of lung cancer patients. The radioresistant lung cancer cells were constructed, and the role of NRF2 in radiation resistance was explored by *in vivo* and *in vitro* experiments. Immunoprecipitation, immunofluorescence and extraction of chromatin fractions were used to explore the underlying mechanisms.

**Results:** In this study, the TCGA database and clinical lung cancer samples showed that high expression of NRF2 was associated with poor prognosis in lung cancer patients. We established radioresistant lung cancer cells expressing NRF2 at high levels, which showed increased antioxidant and DNA repair abilities. In addition, we found that NRF2 can be involved in the DNA damage response independently of its antioxidant function. Mechanistically, we demonstrated that NRF2 promoted the phosphorylation of replication protein A 32 (RPA32), and DNA topoisomerase 2-binding protein 1 (TOPBP1) was recruited to DSB sites in an NRF2-dependent manner.

**Conclusion:** This study explored the novel role of NRF2 in promoting radiation resistance by cooperating with RPA32 and TOPBP1 to activate the ATR-CHK1 signaling pathway. In addition, the findings of this study not only provide novel insights into the molecular mechanisms underlying the radiation resistance of lung cancer cells but also validate NRF2 as a potential target for radiotherapy.

## Introduction

Lung cancer has become the most lethal malignancy, with more than 350 deaths each day 1. Clinical studies showed that approximately 70% of patients need radiotherapy (RT), and approximately 77% of patients with lung cancer receive RT 2. Despite the significant improvement in the efficacy of RT, radiation resistance is a key driver for recurrence. Such as, cancer cells develop resistance during continuous exposure to ionizing radiation (IR) 3. Hence, how lung cancer cells develop radiation resistance and escape the lethal effects of radiation needs to be understood.

Among various types of IR-induced DNA damage, double-strand breaks (DSBs) are the principal mode by which radiation kills tumor cells 4,5. The two major DSB repair mechanisms are homologous recombination (HR) and nonhomologous end joining (NHEJ) 6. HR is the exact repair pathway between two homologous sequences, and therefore, it occurs primarily in the S and G2 phases of the cell cycle 4. The ATM and Rad3-related (ATR)-checkpoint kinase 1 (CHK1) pathway hinders origin firing and mitotic entry by inhibiting cyclin-dependent kinase 2 (CDK2) and CDK1 activity. Thus, ATR promotes HR by regulating G2 cell cycle arrest 7,8. Replication protein A (RPA), a heterotrimer composed of three subunits, RPA70, RPA32, and RPA14, plays an important role in the activation of ATR 9. In cells with DSBs, ATR is recruited to RPA-coated single-strand DNA (ssDNA) stretches through its partner protein ATR-interacting protein (ATRIP) 10. DNA topoisomerase 2-binding protein 1 (TOPBP1) promotes ATR activation when it is recruited to damaged DNA by binding to the C-terminal tail of the RAD9 subunit of the RAD9-HUS1-RAD1 (9-1-1) complex 10. Unlike TOPBP1, Ewing's tumor-associated antigen 1 (ETAA1) is recruited to ssDNA by directly interacting with RPA 9-11. Subsequently, activating proteins, such as TOPBP1 and ETAA1, directly activate ATR by inducing conformational changes in the ATR kinase domain through the ATR activation domain (AAD) 8,10,11.

Nuclear factor erythroid 2-related factor 2 (NRF2), originally isolated as a homolog of hematopoietic nuclear factor erythroid 2 p45, was subsequently discovered as a cancer marker with functions such as maintenance of redox homeostasis and regulation of metabolism 12,13. When cells are exposed to oxidative, electrophilic, or exogenous stress, NRF2 escapes Kelch ECH-associated protein 1 (KEAP1)-dependent repression, translocates into the nucleus, and regulates the transcription of many antioxidant genes containing antioxidant response elements (AREs) 13,14. Previous studies have shown that perturbation of NRF2 pathway is involved in preeclampsia, central nervous system injury, multiple cancer processes, chemotherapy-resistance and RT-resistance. The imbalance of oxidative homeostasis caused by abnormal KEAP1/NRF2 signaling pathway is an important reason for the occurrence of these diseases 15-19. However, recent studies reported that NRF2 not only regulates the redox equilibrium but also adjusts DNA damage repair 20,21. For instance, NRF2 can activate the expression of p53-binding protein 1 (53BP1), which subsequently translocates to DSB sites and promotes NHEJ 12,22.

We previously reported that NRF2 acted as an ATR activator to protect cells against DSBs in a manner independent of its role in antioxidant defense 23. NRF2 deficiency results in the release of G2 cell cycle arrest, impaired HR, elevated levels of apoptosis and micronuclei, and hypersensitivity of cells and xenografts to IR 23,24. However, the detailed mechanism of NRF2 regulating DNA repair process remains to be clarified. In this study, we demonstrated that NRF2 activation caused by NRF2/KEAP1 mutations was associated with poor prognosis in patients with lung cancer. High expression of NRF2 is significant for radiation-resistant cells. NRF2 participated in the DNA damage response (DDR) process in a way independent of transcriptional function. NRF2 enhanced cellular radiation resistance by promoting RPA32 phosphorylation and recruiting RPA and TOPBP1 to DNA damage sites.

## Materials and methods

### Cell culture and drug treatment

The cell lines A549, H460 and H1299 were purchased from the American Type Culture Collection (ATCC, USA) and were cultured in RPMI 1640 (HyClone, USA) supplemented with 10% fetal bovine serum (FBS) (Gibco, New Zealand). U2OS cells were purchased from ATCC and cultured in DMEM (HyClone, USA) supplemented with 10% FBS. The construction process of A549-NRF2^KO^ cells has been described in a previous research article 23. Camptothecin (CPT) was dissolved in dimethyl sulfoxide (DMSO) to a storage concentration of 10 mM and diluted with culture media to a working concentration of 30 nM or 60 nM.

### TCGA and c-BioPortal databases

We searched for the genetic change analysis of NRF2 and KEAP1 on the c-BioPortal website (https://www.cbioportal.org/), which is an online open-access resource. c-BioPortal was accessed in 29 studies (Table S1). DNA copy number alterations, gene expression, and mRNA expression z scores (RNA-seq V2 RSEM) were determined to investigate NRF2 and KEAP1 alterations. The influence of NRF2 on the prognosis of lung cancer was analyzed using Kaplan-Meier plotter. All operations were performed based on c-BioPortal's online instructions.

### Tissue microarray

The immunohistochemical chips for LUSC (98 patients) and LUAD (90 patients) were provided by Shanghai Outdo Biotech Company (XT19-041 or XT19-019, China). The lung cancer tissues were fixed with formalin, and the tissue diameter of the tissue chip array was 1.5 mm. The antibody used for immunohistochemical analysis was anti-NRF2 (Proteintech, 16396-AP). Patients were divided into different groups based on the NRF2 staining intensity in cytoplasm or nucleus, and then the difference in the life span of different groups was determined.

### Radiation-resistant cell line construction

We constructed three radiation-resistant lung cancer cell lines, including A549R, H460R, and H1299R, by exposure to IR repeatedly. When cells reached to approximately 60% confluence in a 10cm dish, they were exposed to ^137^Cs-γ-ray radiation (2 Gy). Then, cells were back to culture and passed as usual. Around one week (6 ~ 9 days) after IR, cells were seeded in a dish and another 2 Gy dose of ^137^Cs -γ-ray radiation was applied at the cell density of ~60%. This process was repeated until the cumulative irradiation dose reached 20 Gy. Finally, the radiation resistance of the cells was determined using the colony formation assay.

### Whole-cell lysate collection and Western blotting

The cells were collected into a 1.5 ml EP tube with trypsin. RIPA lysis buffer (50 mM Tris pH-7.4, 150 mM NaCl, 1% Triton X-100, 1% sodium deoxycholate, 0.1% SDS) was added, then Lysates were supplemented with 4× loading buffer (P0731, Beyotime, China) and heated at 100 °C for 10 minutes. Separation gels and concentrated gels were prepared according to the molecular kDa of the target protein. The samples of the standard (Protein Maker) and experimental groups were added in turn, SDS‒PAGE electrophoresis was carried out, and the target protein was transferred to a PVDF membrane. The antibody diluted with 1% BSA was incubated. Super SigmaTM West Pico PLΜS Chemiluminescent Substrate (Thermo Fisher, 34577). Antibodies and dilution ratios are listed in Table S3.

### Immunofluorescence assays

The cells were inoculated into a Petri dish with preplaced slides. After the cells firmly adhered to the wall, the slide was removed and washed twice with PBS. Cells were fixed with 4% paraformaldehyde (P1110, Solarbio, China) and washed twice with PBS. Next, the cells were treated with 0.5% Triton X-100 (T8200, Solarbio, China) for 20-30 min and washed twice with PBS. The primary antibody was incubated at 4 °C for 24 h, and the secondary antibody was incubated at room temperature for 2 h. Finally, DAPI (Vector Laboratories, H-1500) was used for nuclear staining, and fluorescence changes were observed by fluorescence microscopy. Antibodies and dilution ratios are listed in Table S3.

### Co-immunoprecipitation assays

Cells were collected and washed with PBS for one time. Then they were lysated in NP40 lysis buffer on ice for 30 min, and then centrifuged at 12,000 rpm for 30 min. The antibody and protein A/G beads (B23202; Bimake, Houston USA) were mixed in proportion and then rotated on a shaking table at room temperature for 1 h. Next, the mixture was added to the cell lysate and shaken slowly at 4 °C for incubation overnight. Finally, the bead mixture was centrifuged at 14,000 rpm for 5 s. The protein A/G beads and the antigen-antibody mixtures were collected and rinsed three times with phosphate-buffered saline (PBS). The mixture of protein and beads was added to 2 × loading buffer and heated at 100 °C for 10 min for the Western blotting experiment.

### Measurement of ROS levels

ROS were detected using the fluorescent probe DCFH-DA with an ROS assay kit (88-5930-74; Thermo Fisher Scientific, USA). Then, 40 μL of DMSO was dissolved in ROS assay stain in a 500× storage solution and stored at -20 °C following the manufacturer's protocols. Furthermore, 100 μL of ROS assay buffer containing 1× ROS assay stain was added to the cell culture medium and incubated at 37 °C for 1 h. Finally, the cells were collected, and the changes in the fluorescence signal at 488 nm were detected by flow cytometry.

### Colony formation assay

The cells were inoculated in a six-well plate with 600 cells per well, and 2 mL of the culture medium was added. Next, the cells were irradiated with different doses of ^137^Cs γ-rays. After irradiation, the cells were placed in an incubator at 37 °C for 7 days until colonies (cell numbers >50) were formed. Finally, the colonies were stained with 0.5% crystal violet (C0121, Beyotime, China) for 10 min, and the colony formation ability of cells after irradiation was evaluated by counting the number of colonies.

### Tumor sphere formation assay

The cells in good growth conditions were collected, resuspended in 20 μL of medium, and transferred to a six-well plate with low adhesion (#3471, Costar; China). Serum-free RPMI-1640 containing B27 (1:50), EGF (250 μg/mL), FGF (100 μg/mL), and insulin (1.5 mg/mL) was prepared, 1 mL of which was added to each well of the six-well plate. The cells were placed in a CO_2_ incubator at 37 °C for 3 days. The number of free cell colonies was counted under a microscope.

### Nuclear and cytoplasmic protein extraction

The nuclear and cytoplasmic extraction reagents (78833, Thermo Fisher Scientific) were used for this experiment. The cultured cells were collected and washed twice with PBS. After centrifugation at 2,000 rpm for 15 min, the supernatant was removed, and precooled CER I was added according to the volume of cell precipitation. The tube was oscillated for 15 s and placed at 4 °C for 10 min. Precooled CER II was added to the tube and centrifuged at 12,000 rpm for 10 min. Cytoplasmic protein lysates were included in the supernatant. NER was added to the remaining pellet, which was centrifuged at 12,000 rpm for 10 min after 40 min on ice. The final supernatant contained the nuclear protein lysate.

### Extraction of chromatin fractions

The cultured cells were collected, mixed with NETN lysis buffer (100 mM NaCl; 0.5% NP40; 20 mM Tris-HCl; 1 mM EDTA), and incubated at 4 °C for 30 min. Three vortex oscillations were needed during this period. The cell lysate was then centrifuged at 12,000 rpm for 10 min, and 4× loading buffer was added to the supernatant containing cytoplasm-related proteins and incubated at 100 °C for 10 min. Then, we discarded the excess supernatant and washed the cellular precipitate at the bottom of EP tube three times with PBS. EBC2 (300 mM NaCl, 5 mM CaCl_2_, 500 mM Tris-HCl, 10 U micrococcal nuclease) was added and lysed at 4 °C for 20 min. Finally, the lysate was centrifuged at 14,000 rpm for 15 min and the supernatant contained chromatin-bound proteins.

### Lentiviral transfection experiment

The cells were added to a six-well plate at a density of 1 × 10^5^ per well until the cells grew to 50% confluence. The original medium was replaced with 2 mL of fresh medium containing polybrene (0.5 μg/mL) (C0351, Beyotime). An appropriate amount of viral suspension was added and incubated in an incubator at 37 °C for 4 h. Finally, the culture medium containing the virus was replaced with fresh culture medium, and the cells were continuously cultured for 24 h.

### siRNA transfection experiment

The siRNAs were synthesized by GenePharma Co., Ltd. (Shanghai, China). The sense primer sequences are shown in Table S2. The cells were added to a six-well plate at a density of 1× 10^5^ per well until the cells grew to 50% confluence. The OPTI-MEM (31985070, Thermo Fisher Scientific) medium containing siRNA and RNAiMax (13778075, Thermo Fisher Scientific) was allowed to stand for 15 min before adding the mixture to the medium. After 8 h, the culture medium containing siRNA was replaced with fresh culture medium containing serum. The knockdown effect of the siRNA target gene was detected after 24 and 48 h.

### Plasmid transfection experiment

We used restriction endonucleases to cut the target gene and vector DNA and finally used DNA ligase to connect them together. Then, 2 μg of plasmid and 5 μL of Lipofectamine 2000 (11668019, Thermo Fisher Scientific) were diluted with 100 μL of OPTI-MEM, mixed gently, and placed at room temperature for 20 min. The transfection reagent was added to the culture medium in each well of the six-well plate and then replaced with fresh culture medium after 6 h. The transfection effect of the plasmid was detected after 24 and 48 h.

### Animal experiments

A549 and A549R cells were infected with lentivirus expressing GFP and injected into nude mice via the tail vein. The injection volume of each nude mouse was 5 × 10^6^ cells/150 μL. Approximately 6 weeks later, the nude mouse lung metastasis model was successfully established. The nude mouse lung was irradiated with 2 Gy, 4 Gy, and 2 Gy of γ-rays respectively, once a week. The animals were sacrificed by dislocating the cervical vertebrae, and their lung tissues were removed. The distribution of lung tumors was observed using a fluorescence imaging system. The animal experiment was approved by the Animal Ethical and Welfare Committee of the Institute of Radiation Medicine of Peking Union Medical College (IRM-DWLL-202201).

### Immunohistochemical staining

The lung tissue was embedded in paraffin wax and cut into 4-μm-thick slices, which were fixed on a glass slide. The paraffin-embedded sections were repaired for antigen and then incubated with 3%-5% H_2_O_2_ at room temperature for 10 min to block endogenous peroxidase. The sections were incubated overnight with primary antibody at 4 °C. The paraffin sections were moved from 4 °C to room temperature and incubated with the secondary antibody at 4 °C for 60 min. The DAB substrate kit (ab64238, Abcam) was used to stain the target protein in paraffin-embedded sections, and then the nuclei were restained with hematoxylin. After dehydration with xylene, the tissue slices were dried, neutral resin was dropped on the slides, and the slides were covered with cover glass.

### TUNEL assay

In this experiment, a one-step TUNEL kit (C1089, Beyotime) was used to detect changes in apoptosis. The paraffin-embedded lung sections were dewaxed in xylene for 5-10 min and then washed with PBS three times. The sections were incubated in 3% hydrogen peroxide solution (3% H_2_O_2_) at room temperature for 20 min to inactivate endogenous peroxidase. Then, 50 μL of biotin labeling solution (TdT enzyme: 5 μL; fluorescent labeling solution: 45 μL) was added to the sections and incubated at 37 °C for 60 min in the dark. Finally, the paraffin slices were sealed with DAPI and observed under a fluorescence microscope.

### Statistical analysis

All statistical analyses were performed using the two-tailed Student's *t* test with GraphPad Prism 8.0. All data are expressed as the mean ± S.E.M. We used the log-rank test in univariate survival analyses. All statistical tests were two tailed, and a *P* value <0.05 indicated a statistically significant difference (^*^P < 0.05, ^**^P < 0.01, ^***^P < 0.001, ns: no significance).

## Results

### NRF2 activation caused by *NRF2/KEAP1* gene alteration was correlated with poor prognosis in patients with lung cancer

**Figure 1:**
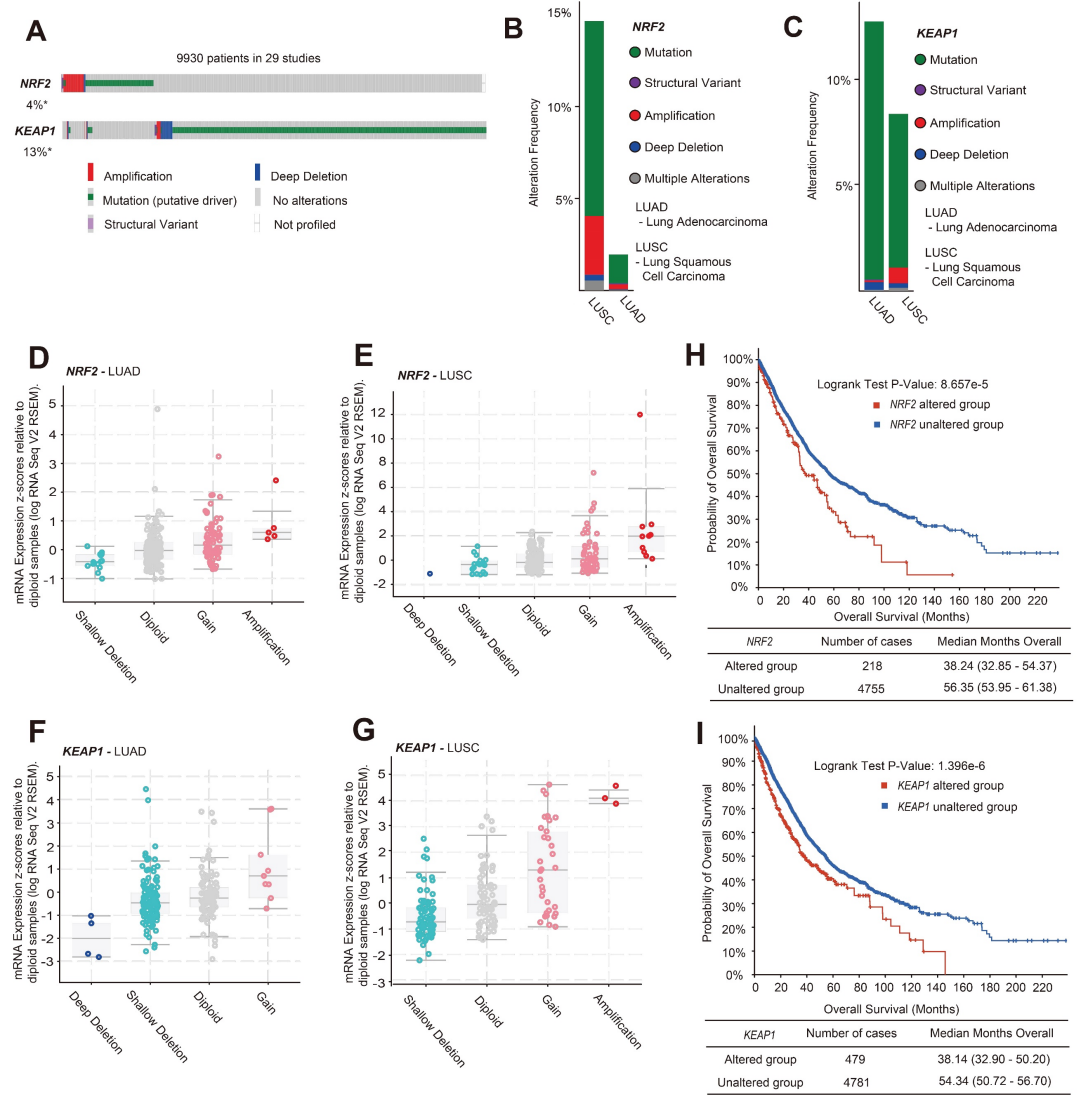
** NRF2 overexpression caused by *NRF2/KEAP1* variation was associated with poor prognosis in patients with lung cancer. A** Gene alteration analysis of *NRF2/KEAP1* in 9,930 lung cancer patients from 29 studies (Table S1) using c-BioPortal online analysis tools. **B, C** Analysis of *NRF2* alteration types (B) and *KEAP1* alteration types (C) in LUSC and LUAD. The number of single cohort cases analyzed for *NRF2* was over 400, and the number of single cohort cases analyzed for *KEAP1* was over 500. **D, E** Plot showing the relationship between *NRF2* mRNA expression and CNV alteration types in LUAD and LUSC (TCGA, Firehose Legacy). **F, G** Plot showing the relationship between *KEAP1* mRNA expression and CNV alteration types in LUAD and LUSC (TCGA, Firehose Legacy). **H, I** Analysis of overall survival (months) corresponding to patients with lung cancer having *NRF2* alterations (H) and *KEAP1* alterations (I).

Current evidence suggests that mutations in the *NRF2/KEAP1* gene may lead to poor prognosis in patients with lung cancer, such as resistance to radiotherapy and chemotherapy, but more specific evidence is needed to validate this 25,26. We analyzed the variant types of the* NRF2/KEAP1* gene in patients with lung cancer through the c-BioPortal website [data from The Cancer Genome Atlas (TCGA) database]. After analyzing 12,478 samples / 9,930 patients with lung cancer from 29 studies, it was found that the total variation in *NRF2* and *KEAP1* genes was 4% and 13%, respectively (Figure 1A). Genetic mutations are the main form of variation in the *NRF2 and KEAP1* genes. Among the copy number variation (CNV) types, amplification was the predominant type of alteration for *NRF2*, while the dominant type of alteration for *KEAP1* was deep deletion (Figure 1A). Different subtypes of lung cancer, including lung adenocarcinoma (LUAD) and lung squamous cell carcinoma (LUSC), have different cell variant types, leading to different growth characteristics 27. Furthermore, we found that the amplification and mutation of *NRF2* mainly occurred in LUSC (Figure 1B). However, compared to LUSC, *KEAP1* gene variants occur more frequently in LUAD, and the main CNV changes are deep deletions (Figure 1C). Next, RNA-seq analysis (TCGA, Firehose Legacy) showed that the main CNV types of *NRF2* in LUAD and LUSC were amplification and gain, which led to an increase in *NRF2* mRNA expression (Figure 1D-E). The main variant type of *KEAP1* was shallow deletion, accompanied by lower *KEAP1* mRNA expression (Figure 1F-G). Furthermore, we investigated the clinical outcomes between patients in the *NRF2/KEAP1* gene-altered and *NRF2/KEAP1* gene-unaltered groups. The results showed that genetic alterations in the *NRF2/KEAP1* gene were associated with shorter overall survival (Figure 1H-I). The median overall survival of patients with *NRF2/KEAP1* alterations was 38.24 and 38.14 months, respectively, while the no gene alterations group had 56.35 and 54.34 months, respectively. Thus, the aforementioned results indicated that the *NRF2/KEAP1* gene variation led to an increase in the level of NRF2 expression and was related to poor prognosis in patients with lung cancer.

**Figure 2:**
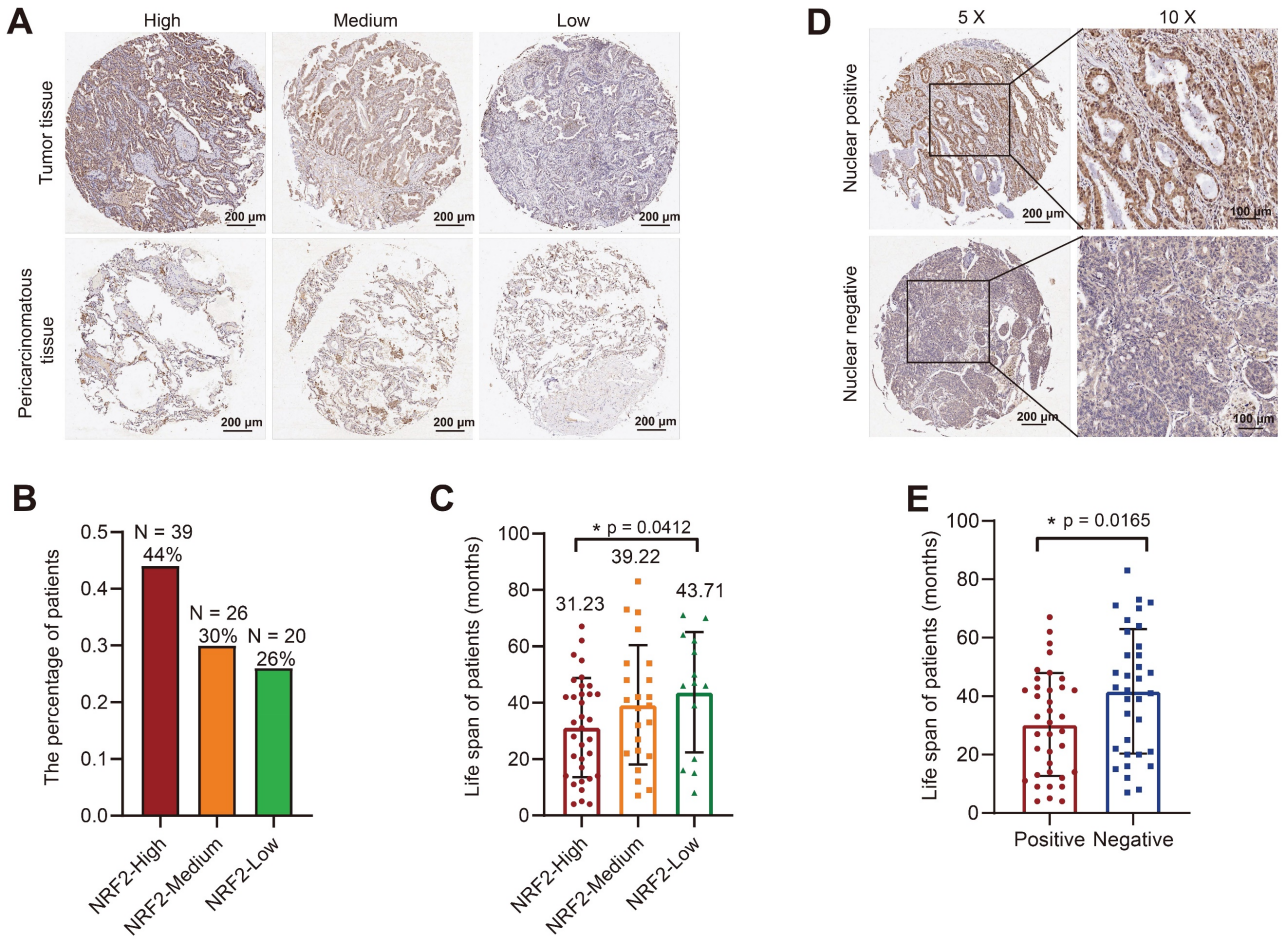
**Correlation analysis of tissue microarray staining for NRF2 and prognosis in patients with LUAD. A** Patients were categorized into three groups, high/medium/low, based on the staining intensity of cytoplasmic NRF2 in the LUAD tissue microarray (representative IHC images of NRF2 are shown). Scale bar, 200 μm. **B** Proportion of patients with LUAD in each group with high/medium/low NRF2 protein levels in the cytoplasm. NRF2-high group (*n* = 39, ratio 44%); NRF2-medium group (*n* = 26, ratio 30%); NRF2-low group (*n* = 20, ratio 26%). **C** Corresponding life span (months) of patients with LUAD in each group with high/medium/low cytoplasmic NRF2 protein levels. The data are presented as the means ± S.E.M. (*n* > 3). ^*^P < 0.05. **D** Patients were divided into two groups based on the positive/ negative staining of NRF2 protein in the nucleus (representative IHC images of NRF2 are shown). Nuclear positive group (NRF2 positive 30%-100%), Nuclear negative group (NRF2 positive 0%-30%). 5× Scale bar: 200 μm. 10× Scale bar: 100 μm. **E** Corresponding life span (months) of patients with LUAD in each group with positive/negative NRF2 protein expression in nuclei. The data are presented as means ± S.E.M. (*n* > 3). ^*^P < 0.05.

An immunohistochemical (IHC) assay was performed to examine the expression of NRF2 protein in the tumor microarray (TMA) composed of lung cancer tissue samples from patients diagnosed with LUSC or LUAD and adjacent noncancerous tissues as controls. We used an NRF2 antibody to stain the TMA and evaluated the score based on the staining intensity. We divided LUSC and LUAD samples into three groups with high/medium/low NRF2 expression levels based on NRF2 staining intensity in cytoplasm (Figure 2A and Figure S1A). In the LUAD group, 44% of patients were in the NRF2 high-expression group (Figure 2B), and the median life span of this group of patients was 31.23 months, which was significantly lower than that of the other groups (Figure 2C). Similarly, in the LUSC group, the proportion of patients with high NRF2 expression was 34%, and the median life span was 34.44 months, which was also significantly lower than that in the other groups (Figure S1B-C).

Further, we divided patients into positive and negative groups based on the localization of NRF2 protein in the nucleus. The results of NRF2 nuclear staining classification in LUAD revealed that patients who tested positive for nuclear NRF2 had a significantly shorter life span compared with patients who tested negative (Figure 2D-E).

Overall, the results of TMA were consistent with the TCGA database analysis results, indicating that the high NRF2 protein expression in patients with lung cancer was associated with poor prognosis.

### High expression of NRF2 was significant for radiation-resistant cells

Several reports have highlighted that the NRF2 protein is related to radiation resistance in cancer cells. Hence, we established radiation-resistant cells with human lung cancer cells (A549, H460, and H1299) (Figure S2A). The colony formation results showed that A549R, H460R, and H1299R cells exhibited significantly higher survival levels after 6 Gy of radiation exposure compared with the parental lung cancer cells (Figure S2B-D). This finding suggested that construction of radioresistant lung cancer cells was successful. Next, we used mass spectrometry to analyze the differentially expressed proteins in H1299/H1299R and H460/H460R cells, respectively. In H1299/H1299R cells, we found 422 differentially expressed proteins, 100 of which were downregulated and 322 of which were upregulated. In H460/H460R cells, proteomics analysis identified that 251 proteins significantly differed in abundance, with 134 downregulated and 117 upregulated in H460R cells. Both Gene Ontology (GO) biological process analysis and Kyoto Encyclopedia of Genes and Genomes (KEGG) enrichment analysis showed differential signaling pathway changes in H1299/H1299R and H460/H460R cells (Figure S2E-H). Furthermore, we found that 13 proteins were upregulated in both H1299R and H460R cells (Table. S4). MAPK and STAT3 are key proteins in the metabolic pathway that intersect with the NRF2 antioxidant related signaling pathway network 28. In addition, NQO1 is one of the important targets of NRF2 in regulating oxidative stress 29.

Cancer stem cells with higher DNA repair ability are particularly resistant to RT. Thus, we initially evaluated the correlation between radiation resistance and tumor cell stemness using the tumor stem cell sphere formation assay. The stemness of A549R cells was significantly higher than that of wild-type A549 cells (Figure S3A-C). The stemness of NRF2 knockout A549 cells was significantly lower than that of radiation-resistant and wild-type cells (Figure S3A).

**Figure 3:**
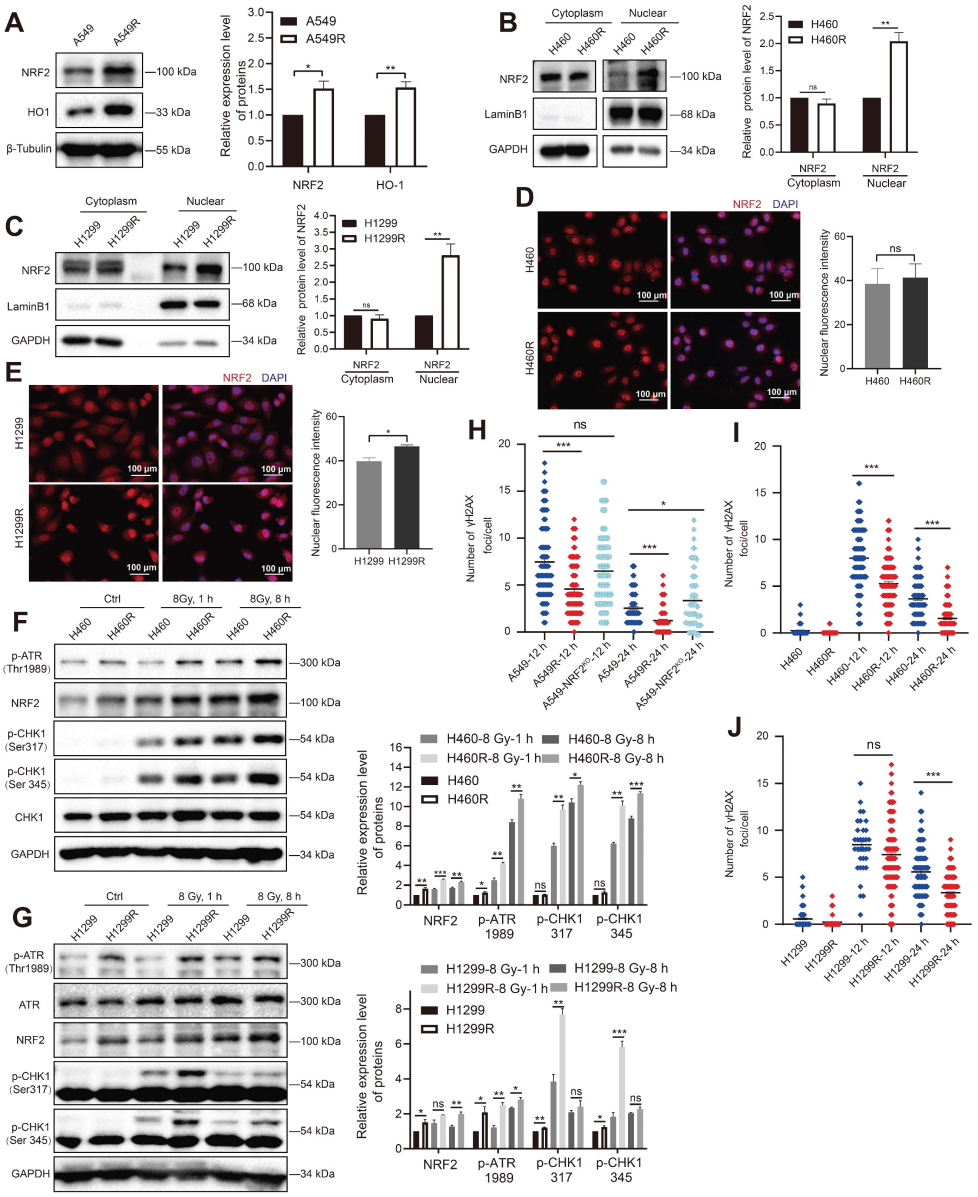
** High NRF2 expression was significant in radiation-resistant cells. A** Western blotting of NRF2 and HO-1 in A549 and A549R cells. **B, C** Western blotting of NRF2 in the nuclear and cytoplasmic extracts of H460/H460R and H1299/H1299R cells. **D, E** Immunofluorescence analysis of NRF2 (red) protein levels in nuclear localization changes in H460/H460R and H1299/H1299R cells. Nuclei were visualized using DAPI staining (blue). Scale bar: 100 μm. **F** Western blotting of NRF2, p-ATR, CHK1, and p-CHK1 in H460 and H460R cells exposed to 8 Gy ^137^Cs γ-rays for 1 and 8 h. **G** Western blotting of NRF2, ATR, p-ATR, and p-CHK1 in H1299 and H1299R cells exposed to 8 Gy ^137^Cs γ-rays for 1 and 8 h. **H-J** A549/A549R/A549-NRF2^KO^, H460/H460R, and H1299/H1299R cells exposed to 4 Gy ^137^Cs γ-rays and γ-H2AX foci were counted 12 and 24 h after irradiation. Data are means ± S.E.M. (*n* > 3). Error bars indicate the standard error of the mean (^*^P < 0.05, ^**^P < 0.01, ^***^P < 0.001, *ns*: no significance).

The generation of reactive oxygen species (ROS) after the ionization of intracellular compounds is one of the main factors leading to DNA damage 30,31. In this study, we used H_2_O_2_ to stimulate oxidative stress in cells to characterize the antioxidant capacity of the constructed radioresistant cells. A549R and H1299R cells showed decreased ROS levels compared with the parental cells, suggesting that the radioresistant cells had an improved ability to remove ROS (Figure S4). As NRF2 is a critical antioxidant regulator, we examined the protein levels of NRF2 in radioresistant cells. We found that the NRF2 protein and its downstream heme oxygenase-1 (HO-1) with antioxidant stress function were significantly upregulated in A549R cells compared with A549 cells (Figure 3A). Furthermore, knocking out NRF2 significantly promoted H_2_O_2_-induced ROS production in A549-NRF2^KO^ cells (Figure S4A), suggesting that the high expression of NRF2 in A549R cells might increase its ability to scavenge ROS.

Translocation to the nucleus is a prerequisite for NRF2 to activate downstream genes that encode an array of phase II detoxifying or antioxidant enzymes 32-34. The nuclear and cytoplasmic separation results showed that the NRF2 protein levels were significantly elevated in H460R and H1299R cells compared with H460 and H1299 cells (Figure 3B-C). Furthermore, the immunofluorescence experiments showed that the NRF2 fluorescence signal was more intense in H1299 radioresistant cells (Figure 3E). These results indicated that NRF2 was upregulated in radiation-resistant cells and preferentially accumulated in the nucleus.

We previously reported that NRF2 led to G2/M cell cycle arrest after DSBs by activating the ATR/CHK1 pathway 23. Next, we monitored the changes in the ATR signaling cascade in radioresistant cells. The Western blotting results showed that IR induced the phosphorylation of ATR and CHK1 in H460 and H1299 cells, and the phosphorylation levels of ATR and CHK1 were obviously higher in H460R and H1299R cells (Figure 3F-G). Consistent with the GO and KEGG analyses of proteomic sequencing, the cell cycle checkpoint proteins of resistant cells underwent changes in H1299R cells (Figure S2E-F). In addition to preventing cells from entering mitosis by activating DNA damage checkpoints, DDR also activates and coordinates various DNA repair pathways. We used an immunofluorescence assay to monitor γ-H2AX foci to assess DNA repair ability, which is an indicator of DNA damage. The numbers of γ-H2AX foci were significantly lower in A549R cells than in A549 cells after exposure to IR, suggesting that the DNA repair ability improved in radioresistant cells compared with control cells (Figure 3H). A similar trend was also observed in H460 and H1299 cells (Figure 3I-J). The aforementioned results indicated that the high levels of NRF2 protein in radioresistant cells enhanced DDR and DNA damage repair capacity, which might be one of the reasons for radiation resistance in lung cancer cells.

### Radiation-resistant cells overexpressing NRF2 were more resistant to radiotherapy *in vivo*

**Figure 4:**
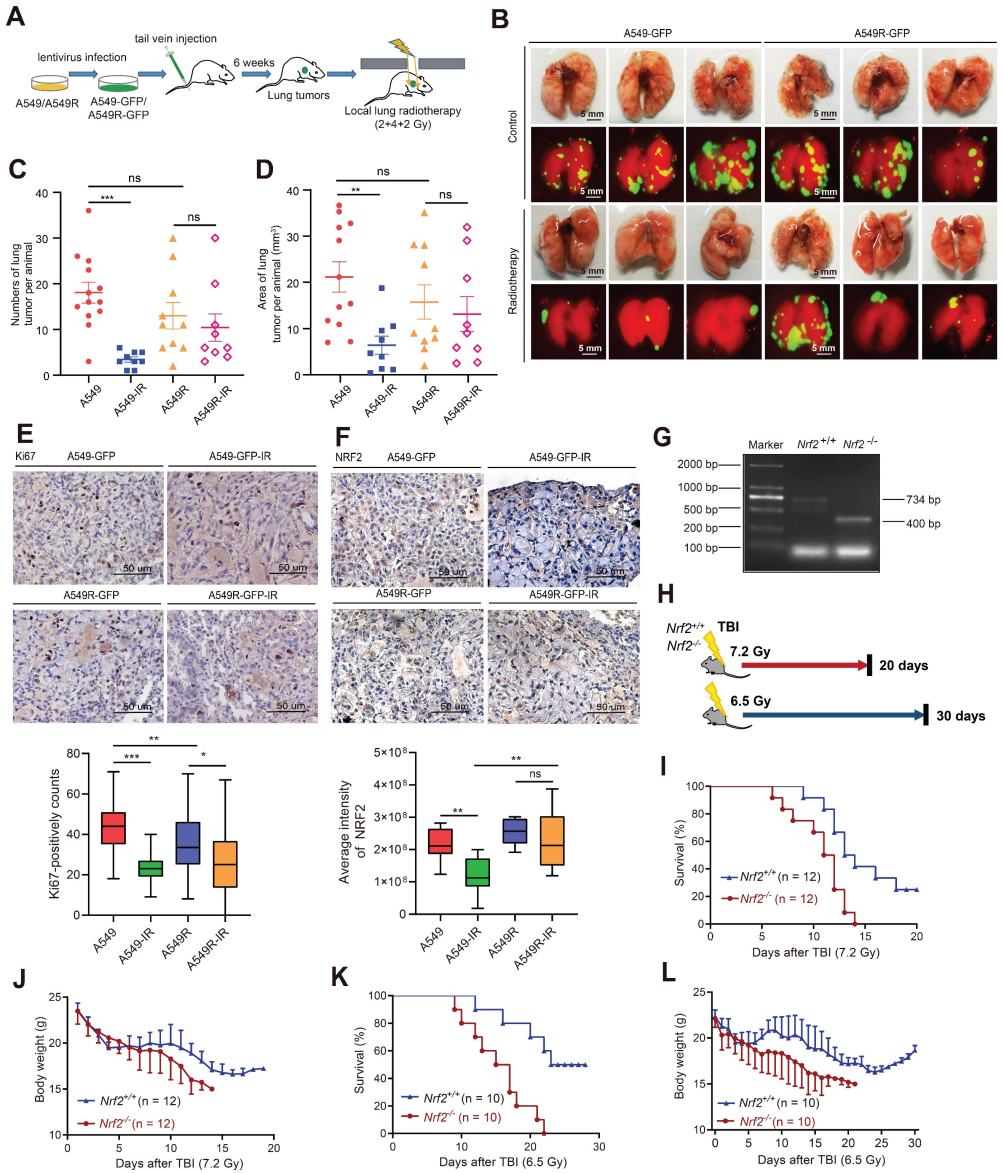
** Radiation-resistant cells with NRF2 overexpression were more resistant to radiotherapy *in vivo*. A** Construction of the lung metastatic tumor model in nude mice. A549-GFP or A549R-GFP cells were injected into nude mice through the tail vein. The metastatic tumor model was successfully constructed after 6 weeks. Then, a total dose of 8 Gy (2, 4 and 2 Gy individually) was used for irradiation, and the interval between each radiation exposure was 1 week. **B** After the nude mice were sacrificed, the lungs were photographed using a fluorescence imaging machine (each group only displays three representative images). Scale bar: 5 mm. **C** Statistics of the number of lung tumors per animal. The data are presented as the means ± S.E.M. (n > 3). ^***^P < 0.001. **D** Statistics of the area of lung tumor per animal (mm^3^). The area was calculated using ImageJ software. The data are presented as means ± S.E.M. (n > 3). ^**^P < 0.01. **E** IHC staining of Ki67 in lung metastases from nude mice. Scale bar: 50 μm.** F** IHC staining of NRF2 in lung metastases from nude mice. Scale bar: 50 μm. **G** *Nrf2*^+/-^ mice (on a C57BL/6J background) were provided by Thomas W. Kensler from the University of Pittsburgh. The genotypes of the mice were determined using polymerase chain reaction. The only band at 734 bp represented the Nrf2^+/+^ mice; the only band at 400 bp represented the *Nrf2*^-/-^ mice.** H** Schematic diagram of the experimental process. **I** Mice were exposed to 7.2 Gy TBI, and the 20-day survival rate was determined (n = 12). **J** Body weight of mice was calculated within 20 days of receiving 7.2 Gy TBI (n = 12). **K** Mice were exposed to 6.5 Gy TBI, and the 30-day survival rate was calculated (n = 10). **L** Body weight of mice was calculated within 30 days of receiving 6.5 Gy TBI (n = 10).

We constructed a lung metastatic tumor model to further confirm the resistance of radiation-resistant cells highly expressing NRF2 protein *in vivo*. A549 and A549R cells stably expressing GFP (Figure S5A-B) were injected into the tail vein of nude mice. After the lung tumor metastasis model was successfully constructed in 6 weeks, the mice were divided into four groups: a control group (A549), a radioresistant cell group (A549R), a control irradiation group (A549-IR), and a radioresistant cell irradiation group (A549R-IR). We locally irradiated the lungs with a 2 or 4 Gy fractionated dose of γ-ray once a week, and the treatment lasted 3 weeks (Figure 4A). We observed tumor metastasis in the lungs with a fluorescence signal (Figure 4B). The number of lung tumor metastases decreased significantly after radiotherapy in the A549-IR group compared with the A549 group. In contrast, no statistically significant difference was observed in the number of lung metastases receiving or not receiving radiotherapy in mice injected with A549R cells, suggesting that the A549R tumors were more resistant to IR (Figure 4C-D). The results showed that A549R cells were also radiation resistant *in vivo*.

IHC analysis of lung tissue sections showed that Ki67-positive staining in A549 cell-derived tumors significantly decreased after irradiation, but the abundance of Ki67 in the A549R-IR group was less different from that in the A549R-unirradiated group, suggesting that IR might not efficiently inhibit the proliferation of A549R cells (Figure 4E). Furthermore, we used IHC to examine the staining intensity of NRF2. The NRF2 staining of the A549 group decreased significantly after radiation, while the change in the A549R group was not significant (Figure 4F). Next, we used a transferase-mediated dUTP-biotin nick end labeling (TUNEL) assay to detect apoptotic cells in lung metastases after irradiation. The percentage of apoptotic cells in metastatic tumors formed by A549 cells was augmented significantly after irradiation. However, the proportion of apoptotic cells in A549R cell metastases did not change significantly after irradiation (Figure S5F). Collectively, these findings indicate an association between NRF2 protein and radiation resistance of lung cancer cells *in vivo*.

Next, we routinely recovered *Nrf2*^+/+^ and *Nrf2*^-/-^ pups from *Nrf2*^+/-^ C57BL/6 intercrosses and determined their genotypes to further explore the role of NRF2 in response to IR *in vivo* (Figure 4G). Then, we exposed them to two doses of IR (7.2 and 6.5 Gy) (Figure 4H). When *Nrf2*^+/+^ and *Nrf2*^-/-^ mice were exposed to 7.2 and 6.5 Gy of IR, *Nrf2*^-/-^ mice exhibited greater mortality and a faster rate of death (Figure 4I and 4K). The average body weight of *Nrf2*^-/-^ mice continued to decrease after total body irradiation (TBI); however, the average body weight of *Nrf2*^+/+^ mice tended to be stable approximately 20 days after TBI (Figure 4J and 4L). These results indicated that deleting the NRF2 gene led to higher mortality rates in mice after radiation exposure, implying that NRF2 might play a protective role in response to IR *in vivo*.

### NRF2 activated the ATR/CHK1 pathway independent of its transcriptional function

**Figure 5:**
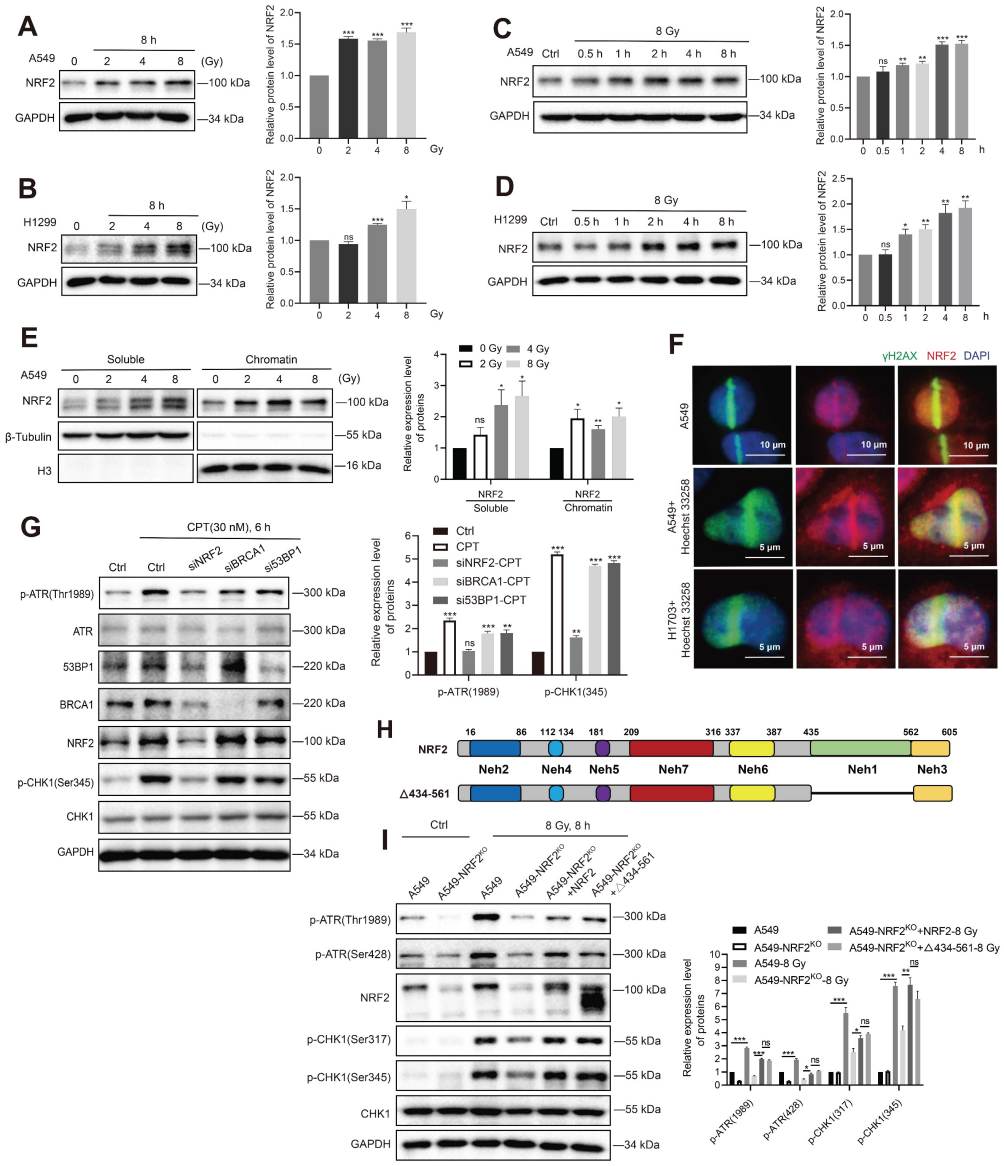
** NRF2 activated the ATR/CHK1 pathway independent of its transcriptional function. A, B** Western blotting of NRF2 in A549 and H1299 cells treated with different doses of ^137^Cs γ-rays (2, 4, 8 Gy) for 8 h. **C, D** Western blotting of NRF2 in A549 and H1299 cells exposed to 8 Gy ^137^Cs γ-rays for different time durations. **E** Western blotting of NRF2 binding to chromatin under damage induced by different doses of ^137^Cs γ-rays (2, 4, 8 Gy) in A549 cells. **F** Immunofluorescence analysis of NRF2 (red) at the site of DNA damage (γ-H2AX: green) in A549 and H1703 cells caused by laser microirradiation. The nuclei were visualized using DAPI staining (blue). Scale bar: 10 μm and 5 μm. **G** Western blotting of ATR, p-ATR, NRF2, 53BP1, BRCA1, CHK1, and p-CHK1 in H1299R cells transfected with siNRF2, siBRCA1, or si53BP1 and treated with 30 nM CPT for 6 h.** H** Schematic illustration of the HA-tagged NRF2 mutants. **I** Western blotting of p-ATR, NRF2, p-CHK1, and CHK1 in A549 cells, A549-NRF2^KO^, A549-NRF2^KO^+NRF2, and A549-NRF2^KO^+Δ434-561-NRF2 exposed to 8 Gy γ-rays for 8 h. Error bars indicate the standard error of the mean, N = 3 independent experiments (*P < 0.05, **P < 0.01, ***P < 0.001, *ns*: no significance).

The aforementioned experiments showed that the elevated NRF2 protein levels in lung cancer cells might be the reason for resistance to radiotherapy. Next, we sought to elucidate the mechanism underlying the contribution of NRF2 to radiation resistance. In A549, H1299, and H1703 cells, irradiation increased the protein level of NRF2 in a dose-dependent manner (Figure 5A-B and Figure S6A); 8 Gy irradiation increased NRF2 protein levels in a time-dependent manner (Figure 5C-D). We found that the accumulation of NRF2 protein in the nuclei of lung cancer tissues was associated with a poorer prognosis of lung cancer (Figure 2D -E), and the level of NRF2 protein in the nuclei of radiation-resistant cells was also higher (Figure 3B-C). Thus, we separated the chromatin from the cells and examined the binding of NRF2 to chromatin after irradiation. The results showed that the binding of NRF2 protein to chromatin increased significantly after irradiation (Figure 5E), suggesting that NRF2 was translocated to the nucleus and bound to chromatin upon IR. Furthermore, we investigated the subcellular localization of NRF2 with immunofluorescence assay. The results illustrated that NRF2 was recruited to DNA damage sites caused by laser microirradiation and colocalized with γ-H2AX in both U2OS, H1703 and A549 cells (Figure 5F and Figure S6B).

As a transcription factor, NRF2 is reported to regulate the expression of BRCA1 and 53BP1 under stress conditions 35,36. Both BRCA1 and 53BP1 are key proteins in the DDR process 37,38. We found that the levels of 53BP1 and BRCA1 proteins decreased significantly after knocking down NRF2 in A549 cells (Figure 5G). However, the small interfering RNA (siRNA)-mediated knockdown of 53BP1 and BRCA1 did not affect the activation of ATR and CHK1 after CPT treatment (Figure 5G). These results indicated that 53BP1 and BRCA1 might not be mediators of NRF2/ATR/CHK1 signaling pathway activation. To confirm NRF2 promoting the activation of ATR-CHK1 signaling pathway is not dependent on its transcriptional regulatory function, we constructed plasmids encoding mutated NRF2 deleted the Neh1 domain (Δ434-561-NRF2) (Figure 5H). Δ434-561-NRF2 lost the ability to regulate transcription. Next, we separately expressed HA-NRF2 and Δ434-561-NRF2 in A549-NRF2^KO^ cells. When the cells were treated with 8 Gy IR, the expression of both HA-NRF2 and Δ434-561-NRF2 significantly promoted ATR and CHK1 phosphorylation (Figure 5I). Altogether, these results suggested that NRF2 could maintain genomic stability independent of transcription function.

### NRF2 promoted the phosphorylation of RPA32 and the accumulation of RPA at ssDNA

**Figure 6:**
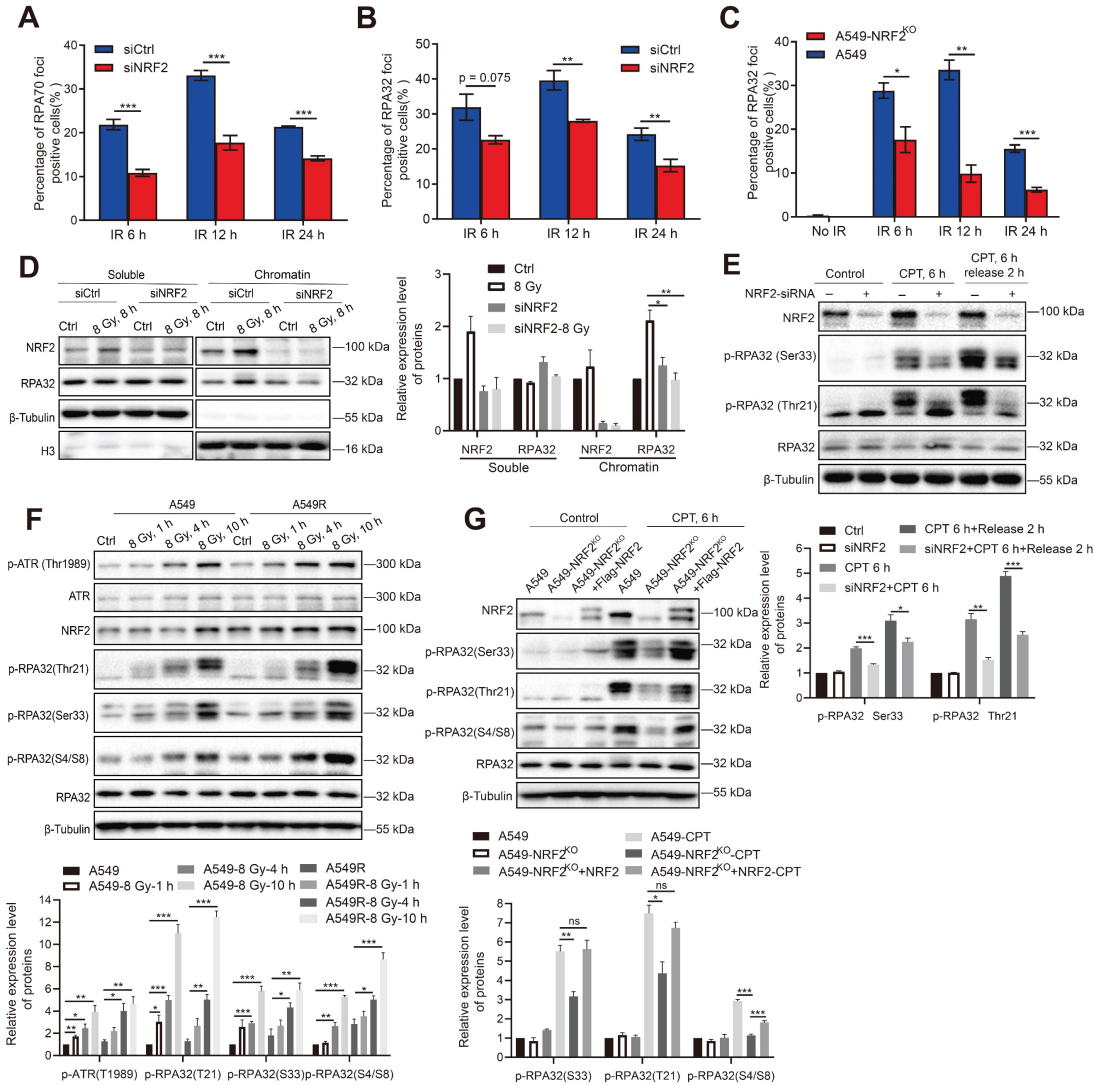
** NRF2 promoted the phosphorylation of RPA32 and the accumulation of RPA at ssDNA. A, B** A549 cells transfected with siNRF2 or siCtrl and exposed to 8 Gy ^137^Cs γ-rays. RPA32 and RPA70 foci were counted 6, 12, and 24 h after irradiation. The data are presented as means ± S.E.M., with *>*150 cells counted in 3 independent experiments. ^***^P < 0.001, ^**^P < 0.01. **C** Immunofluorescence analysis of RPA32 foci in A549 and A549-NRF2^KO^ cells exposed to 8 Gy ^137^Cs γ-rays for 6, 12, and 24 h (γ-H2AX: green; RPA32: red). The data are presented as means ± S.E.M, with *>*150 cells counted in 3 independent experiments. ^*^P < 0.05, ^**^P < 0.01, ^***^P < 0.001. **D** Western blotting of RPA32 and NRF2 proteins in chromatin and soluble extracts of A549 cells transfected with siNRF2 and then exposed to 8 Gy ^137^Cs γ-rays for 8 h. **E** Western blotting of NRF2, p-RPA32, and RPA32 in A549 cells transfected with siNRF2 and then treated with 30 nM CPT for 6 h. **F** Western blotting of p-ATR, ATR, NRF2, p-RPA32, and RPA32 in A549 and A549R cells exposed to 8 Gy γ-rays for 1, 4, and 10 h. **G** Western blotting of NRF2, p-RPA32, and RPA32 in A549, A549-NRF2^KO^, and A549-NRF2^KO^+Flag-NRF2 cells treated with 30 nM CPT for 6 h or 8 Gy ^137^Cs γ-rays for 8 h. Error bars indicate the standard error of the mean (^*^P < 0.05, ^**^P < 0.01, ^***^P < 0.001, *ns*: no significance).

NRF2 can directly bind to and activate ATR, and RPA regulates ATR activation. Hence, we explored whether NRF2 could affect the roles of RPA in the DDR process. RPA70 and RPA32 are the two main subunits of the RPA complex. The number of RPA32 and RPA70 foci was examined 6, 12, and 24 h after irradiation in A549 cells treated or not treated with siNRF2. The immunofluorescence results showed that the proportion of RPA70 foci-positive cells in A549 cells was significantly higher than in A549 cells knocked down NRF2 after they were exposed to IR (Figure 6A). Similarly, knocking down NRF2 and knocking out NRF2 decreased the proportion of RPA32 foci-positive cells after cells were exposed to IR (Figure 6B-C). DNA bound RPA interacts with many proteins to regulate DNA metabolism, and the ssDNA encapsulated by RPA is also a platform for replicating stress-induced DNA damage response processes 39,40. Next, we extracted chromatin-bound proteins to determine whether NRF2 affected the binding of RPA32 proteins to DNA. We found that knockdown of NRF2 markedly inhibited the binding of the RPA32 protein to chromatin after irradiation but did not affect the soluble form of RPA32 (Figure 6D). The results implied that NRF2 might facilitate the binding of RPA32 to chromatin to form foci under DNA damage conditions.

The phosphorylation of RPA32 is a marker of ATR activation 41,42. Hence, we examined whether NRF2 could influence the phosphorylation of RPA32. In A549 cells, knockdown of NRF2 reduced the phosphorylation of RPA at Ser33 and Thr21 after CPT treatment (Figure 6E). Furthermore, we observed a continuous increase in the phosphorylation level of RPA32 (Ser33, Thr21, and S4/S8) within 10 h of irradiation. And the increasing in phosphorylation of RPA32 in A549R cells was more pronounced (Figure 6F). The phosphorylation of RPA32 was markedly lower in NRF2 knockout cells than in wild-type cells, and this defect was rescued by reintroducing Flag-NRF2 into A549-NRF2^KO^ cells after CPT (Figure 6G) or IR treatment (Figure S6C). Hence, our results suggested that NRF2 affected the recruitment of RPA on DNA by promoting the phosphorylation of RPA32 in DDR process.

### NRF2 cooperated with TOPBP1 to activate the ATR/CHK1 signaling pathway

**Figure 7:**
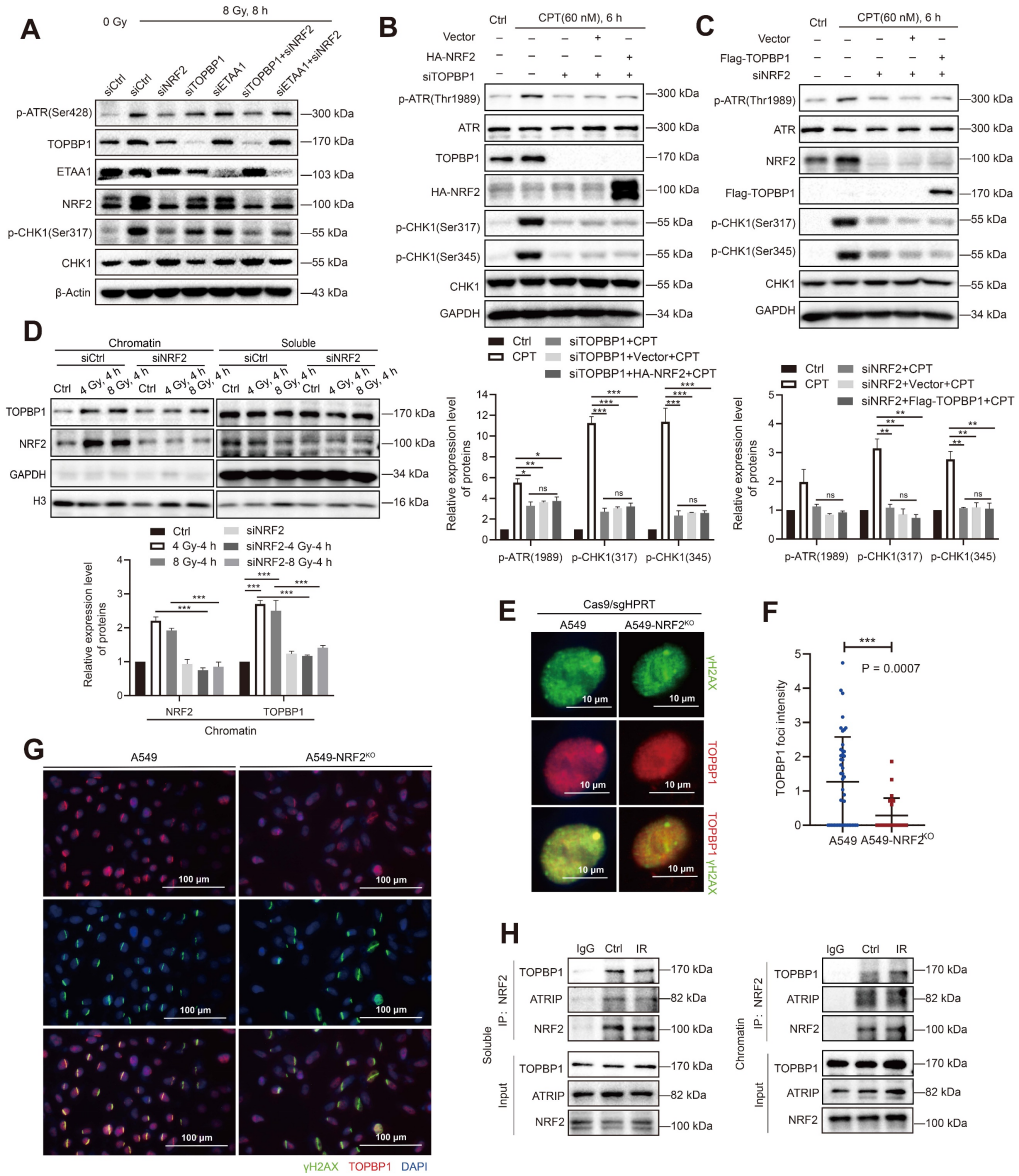
** NRF2 cooperated with TOPBP1 to activate the ATR/CHK1 signaling pathway. A** Western blotting of p-ATR, TOPBP1, NRF2, ETAA1, and p-CHK1 in A549 cells transfected with siNRF2, siTOPBP1, or siETAA1 and then exposed to 8 Gy γ-rays for 8 h. **B** Western blotting of p-ATR, ATR, TOPBP1, HA-NRF2, p-CHK1, and CHK1 in H1299R cells transfected with siTOPBP1 and HA-NRF2 and treated with 60 nM CPT for 6 h. **C** Western blotting of p-ATR, ATR, NRF2, Flag-TOPBP1, p-CHK1, and CHK1 in A549 cells transfected with siNRF2 and Flag-TOPBP1 and treated with 60 nM CPT for 6 h. **D** Western blotting of TOPBP1 and NRF2 in chromatin and soluble extracts of A549R cells transfected with siNRF2 and then exposed to 4 or 8 Gy ^137^Cs γ-rays for 4 h. **E** Immunofluorescence analysis of TOPBP1 (red) in the DNA damage site (γ-H2AX: green) of A549 and A549-NRF2^KO^ cells caused by transfection with Cas/sgHPRT. Scale bar: 10 μm. **F** Statistics of TopBP1 foci intensity. The data are presented as means ± S.E.M. (*n* > 3). ^***^P < 0.001. **G** Immunofluorescence analysis of TOPBP1 (red) at the site of DNA damage (γ-H2AX: green) in A549 cells and A549-NRF2^KO^ cells caused by laser microdissection. Scale bar: 100 μm. **H** Soluble and chromatin extracts of H1299R cells exposed to 8 Gy ^137^Cs γ-rays were subjected to immunoprecipitation with an anti-NRF2 antibody or control immunoglobulin G. Western blotting of TOPBP1, ATRIP, and NRF2 in soluble and chromatin immunoprecipitated extracts. Error bars indicate the standard error of the mean (^*^P < 0.05, ^**^P < 0.01, ^***^P < 0.001, *ns*: no significance).

RPA32 and CHK1 are both phosphorylation substrates of ATR 43. Recent studies indicate that the activation of TOPBP1 and ETAA1 corresponds to different ATR functions 44. ETAA1 is responsible for ATR-mediated RPA phosphorylation during normal and stress replication, while TOPBP1 activates the ATR/CHK1 signaling pathway in response to replication stress 8,45. The results of our study showed that NRF2 not only affected the phosphorylation of RPA32 after DNA damage (Figure 6) but also activated the ATR/CHK1 pathway, leading to G2/M cell cycle arrest 23. Therefore, the overlap between NRF2, ETAA1, and TOPBP1 may indicate the interactions among these proteins. NRF2, TOPBP1, and ETAA1 were individually knocked down by siRNAs in A549 cells to compare the differences between NRF2, TOPBP1, and ETAA1 to activate the ATR/CHK1 pathway. The results indicated that the phosphorylation levels of ATR and CHK1 after 8 Gy irradiation were even more significantly reduced in A549 cells transfected with siNRF2 than in cells transfected with siTOPBP1 and siETAA1, suggesting that NRF2 played a significant role in the phosphorylation of ATR (Figure 7A). Furthermore, we found that the simultaneous knockdown of TOPBP1 and NRF2 did not further lower the ATR phosphorylation level compared with the knockdown of NRF2 alone (Figure 7A). These observations suggested that NRF2 might function with TOPBP1 in the same pathway. We constructed Flag-TOPBP1 and HA-NRF2 fusion proteins and verified their expression in A549 cells to further assess the involvement of NRF2 in the activation of the ATR pathway by TOPBP1 (Figure S6D). Notably, ATR/CHK1 was not phosphorylated in H1299R cells transfected with siTOPBP1 after treatment with 60 nM CPT (Figure 7B). This defect was not rescued by expressing HA-NRF2, indicating the key role of TOPBP1 in the NRF2/ATR/CHK1 pathway (Figure 7B). In parallel, the expression of Flag-TOPBP1 also failed to phosphorylate ATR and CHK1 in NRF2 knockdown A549 cells (Figure 7C). The results suggested that NRF2 and TOPBP1 were not epistatic for ATR activation in DDR.

TOPBP1 is recruited to DNA damage sites through its complex with RAD9-HUS1-RAD1 (9-1-1) and mediates the phosphorylation of ATR through its AAD-like region binding. In chromatin-bound protein detection experiments, Western blotting showed that the downregulation of NRF2 markedly inhibited the binding of TOPBP1 protein to chromatin in irradiated A549 cells (Figure 7D). We used Cas9/sgRNA targeting the HPRT gene to achieve a position-specific DSB to further confirm the role of NRF2 in TOPBP1 recruitment to DNA damage sites. Fluorescence microscopy revealed the colocalization of TOPBP1 with γH2AX in A549 cells. NRF2 deficiency significantly attenuated the intensity of TOPBP1 foci in A549-NRF2^KO^ cells (Figure 7E-F). Normally, NRF2 is modified by KEAP1-mediated ubiquitination and degraded by the proteasome pathway. Additionally, we knocked down KEAP1 to examine the effect of endogenous NRF2 on the recruitment of TOPBP1 to DNA damage sites after irradiation. Among H1299 and U2OS cells, the intensity of TOPBP1 fluorescence was significantly higher in cells with knockdown of KEAP1 than in control cells (Figure S6E-F). Additionally, the results shown in Figure 7G clearly show that TOPBP1 was recruited to DNA damage sites caused by laser micro-radiation in A549 cells but not in A549-NRF2^KO^ cells. These results suggested that NRF2 could affect the recruitment of TOPBP1 to damage sites during DDR. Next, we used co-immunoprecipitation (Co-IP) to verify the interaction between TOPBP1 and NRF2 in the DDR. TOPBP1 was detected after NRF2 was immunoprecipitated from the chromatin and soluble extracts of A549 cells. Moreover, we found that the interaction between TOPBP1 and NRF2 was enhanced in the chromosomal extracts of cells after IR (Figure 7H). These results demonstrated that NRF2 cooperated with TOPBP1 to promote the activation of the ATR/CHK1 signaling pathway and had a promoting effect on the recruitment of TOPBP1 to DNA damage sites following irradiation.

## Discussion

Radiotherapy resistance of tumor cells is a serious issue in the clinical treatment of cancer. Analyses of the TCGA database and lung cancer samples in the TMA demonstrated that NRF2 overexpression was associated with poor prognosis in patients with lung cancer. Moreover, the high expression of NRF2 is one of the reasons for radiation resistance. Importantly, NRF2 senses DNA damage and is recruited to DNA damage sites independent of transcriptional functions. We systematically demonstrated that NRF2 was associated with tumor progression and survival after radiotherapy by overexpressing NRF2 or deleting NRF2 in animal models (Figure 3-4). In this study, NRF2 overexpression and its function in DDR and DNA damage repair were observed in radioresistant lung cancer cells (Figure 3). This notion was confirmed by the construction of a lung metastasis model in nude mice with radiotherapy (Figure 4). In contrast, we found during the construction of the nude mouse lung metastatic tumor model that A549R cells with high expression of NRF2 protein formed fewer lung metastatic tumors than A549 cells injected with the same number of lung cancer cells (Figure 4). Previous studies have shown that the function of NRF2 in different stages of cancer is controversial. NRF2 activation may play a role in avoiding precancerous occurrence in the early stage of tumorigenesis 46,47. Furthermore, why and how NRF2 plays a role in the clinical outcomes of lung cancer needs further exploration.

Previous studies exploring the role of NRF2 in cancer have mainly focused on its transcriptional regulatory function 46,48. Many target genes of NRF2, including redox transcription factors, cell cycle regulators, proliferation regulators, and heme and iron metabolism proteins, may play a role in cancer progression 49,50. However, in this study, we proved that NRF2 participated in the DDR process independent of transcriptional function. In terms of the new function of NRF2, it is expected that lung tumor cells overexpressing NRF2 are resistant to radiotherapy. These results not only provide evidence supporting the role of NRF2 in DDR to radiotherapy but also imply that the nuclear NRF2 expression level might serve as a biomarker for predicting the radiosensitivity of lung cancer.

Mechanistically, previous studies reported that ATR responded to DNA damage by interacting with RPA-covered ssDNA 51. In this study, we found that the depletion of NRF2 significantly reduced the phosphorylation of RPA32 in response to DSBs caused by IR or CPT (Figure 6), implying that NRF2 could mediate RPA32 function. RPA participates in almost all DNA metabolic activities in cells, and its phosphorylation may play a role in regulating these interactions 52. Hyperphosphorylation may alter the structure or conformation of RPA and affect its interaction with ssDNA 53. Therefore, NRF2 may regulate the interaction of RPA with proteins related to DNA damage repair and response signaling pathways by influencing the hyperphosphorylation of RPA32. Previous studies reported that the phosphorylation of RPA32 hindered the association between the RPA complex and cell replication function; additionally, the phosphorylation of RPA stimulated DNA repair and promoted the protection and recovery of the replication fork after genotoxic damage 41,54,55. This result suggested a functional transition from DNA replication to DNA damage signal transduction and repair. The effect of phosphorylation on the interaction between RPA and ssDNA remains controversial 56. Additionally, whether NRF2 phosphorylates RPA32 through ATR-mediated phosphorylation or directly phosphorylates RPA32 needs further verification.

The activation of CHK1 and RPA32 by ATR requires different amounts of RPA ssDNA to coordinate checkpoint activation and DNA repair at DNA damage sites, regulating different DNA repair events 57. Although ATR phosphorylates RPA32 and CHK1 in different ways, it may also occur in different stages of the same process. Compared with ETAA1, TOPBP1 is more essential in activating the CHK1 signaling pathway 11. The N-terminus of TOPBP1 needs to form a stable complex on RPA ssDNA to effectively activate ATR 58. For example, NBS1 is necessary for TOPBP1 to recruit DNA replication-stagnated sites and directly activates ATR independently of MRE11 and TOPBP1 59. Studies on the relationship between TOPBP1 and NRF2 are lacking. In this study, we first revealed that NRF2 was translocated to the nucleus and aggregated at DNA damage sites, promoting the recruitment of TOPBP1 to DNA damage sites; additionally, NRF2 cooperated with TOPBP1 to promote the activation of the ATR/CHK1 signaling pathway (Figure 7). Interestingly, coupled with the effect of NRF2 on RPA32 phosphorylation under stress conditions, NRF2 was involved in the activation of all downstream events related to ATR. Moreover, the knockdown of NRF2 had a greater impact on ATR phosphorylation than the knockdown of ETAA1 and TOPBP1 upon IR, indicating that NRF2 might be the most important activating factor for ATR signaling in response to γ-radiation. Furthermore, our results supported the hypothesis that RPA32 and CHK1 might respond to DNA damage in different stages. Nevertheless, how NRF2 is recruited to DNA damage sites and how functional interactions occur between NRF2 and TOPBP1 in response to IR remain to be established.

**Figure 8:**
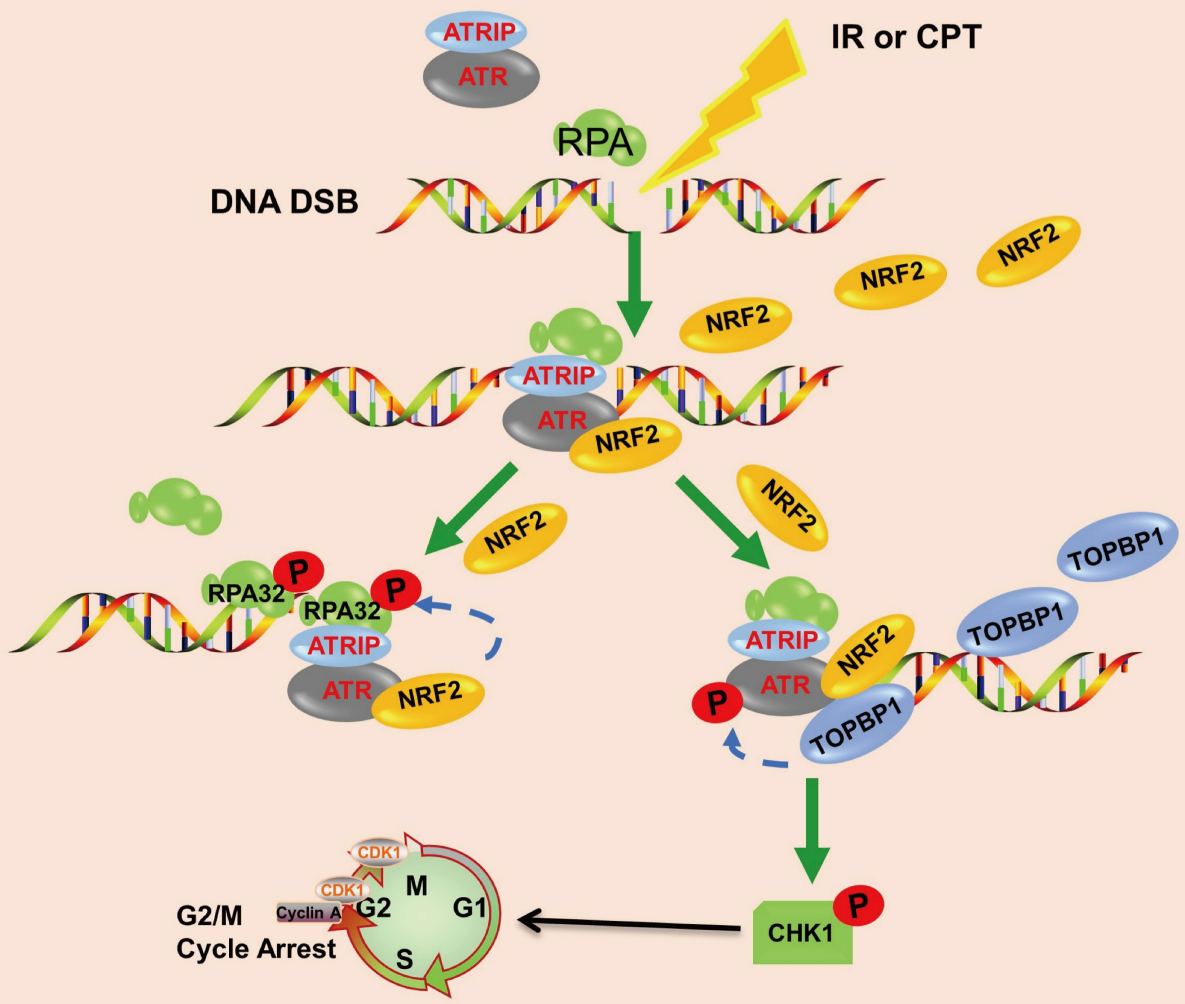
Schematic model showing that NRF2 was translocated to DSB sites, promoted the phosphorylation of RPA32, and activated the ATR/CHK1 pathway by recruiting TOPBP1 to DNA damage sites.

In conclusion, we demonstrated that the high nuclear expression of NRF2 was correlated with poor prognosis in patients with lung cancer and contributed to the development of radiation resistance. In response to IR or CPT, NRF2 was translocated to the DSB sites, promoted the phosphorylation of RPA32, and activated the ATR/CHK1 pathway by recruiting TOPBP1 to the DNA damage sites (Figure 8). This study suggested that NRF2 might be a promising target for improving lung cancer radiotherapy.

## Supplementary Material

Supplementary figures and tables.

## Abbreviations

AAD: ATR activation domain
ARE: antioxidant response elements
CNV: copy number variation
DDR: DNA damage response
DSB: double strand breaks
FBS: fetal bovine serum
HR: homologous recombination
IR: ionizing radiation
LUAD: lung adenocarcinoma
LUSC: lung squamous cell carcinoma
NHEJ: non-homologous end joining
ROS: reactive oxygen species
RT: radiotherapy
TMA: tumor microarray
