# Supplementary material for: NRF2 promotes radiation resistance by cooperating with TOPBP1 to activate the ATR-CHK1 signaling pathway
Source: Theranostics. 2024 Jan 1;14(2):681–98. doi: 10.7150/thno.88899 (PMC10758056; doi:10.7150/thno.88899)
Supplement: Supplementary file 1 — Supplementary figures and tables. [file thnov14p0681s1.pdf]

Supplementary materials for

NRF2 promotes radiation resistance by cooperating with TOPBP1 to activate the ATR–CHK1 signaling pathway

Xiaohui Sun[1#], Mingxin Dong[1#], Jiale Li[1], Yuxiao Sun[1], Yu Gao[1], Yan Wang[1], Liqing Du[1*], Yang Liu[1], Kaihua Ji[1], Ningning He[1], Jinhan Wang[1], Manman Zhang[1], Huijuan Song[1], Chang Xu[1*], Qiang Liu[1*]

Correspondence to:

Liqing Du (dlq@irm-cams.ac.cn)

Chang Xu (xuchang@irm-cams.ac.cn)

Qiang Liu (liuqiang@irm-cams.ac.cn)

This PDF file includes:

Figure. S1 to S6;

Table. S1 and S4

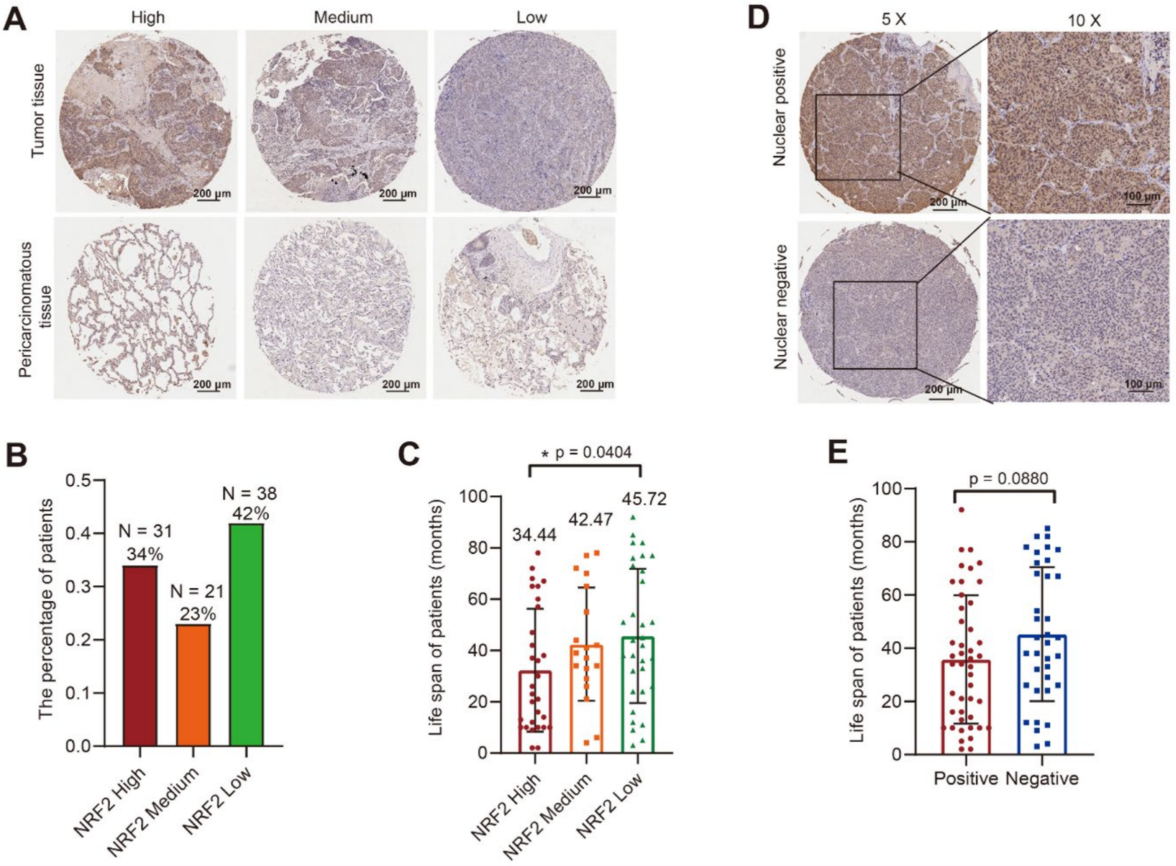

Figure S1. Correlation analysis of tissue microarray stained with NRF2 and prognosis in patients with LUSC.

A Patients were categorized into three groups, high/medium/low, based on the staining intensity of cytoplasmic NRF2 in the LUSC tissue microarray (representative IHC images of NRF2 are shown). Scale bar: 200 μm. **B** Proportion of patients with LUSC in each group with high/medium/low NRF2 protein levels in the cytoplasm. NRF2-high group (n = 31, Ratio 34%); NRF2-medium group (n = 21, Ratio 23%); NRF2-low group (n = 38, Ratio 42%). **C** Corresponding life span (months) of LUSC patients in each group with high/medium/low cytoplasmic NRF2 protein levels. The data are presented as the means ± S.E.M. $^{*}P < 0.05$. **D** Patients were divided into two groups based on the positive/ negative staining of NRF2 protein in the nucleus (representative IHC images of NRF2 are shown). Nuclear positive group

(positive NRF2: 30%-100%), Nuclear negative group (positive NRF2: 0%-30%). 5× Scale bar: 200 μm. 10× Scale bar: 100 μm. **E** Corresponding life span (months) of LUSC patients in each group with positive/negative NRF2 protein in nuclei. Data are presented as the means ± S.E.M.

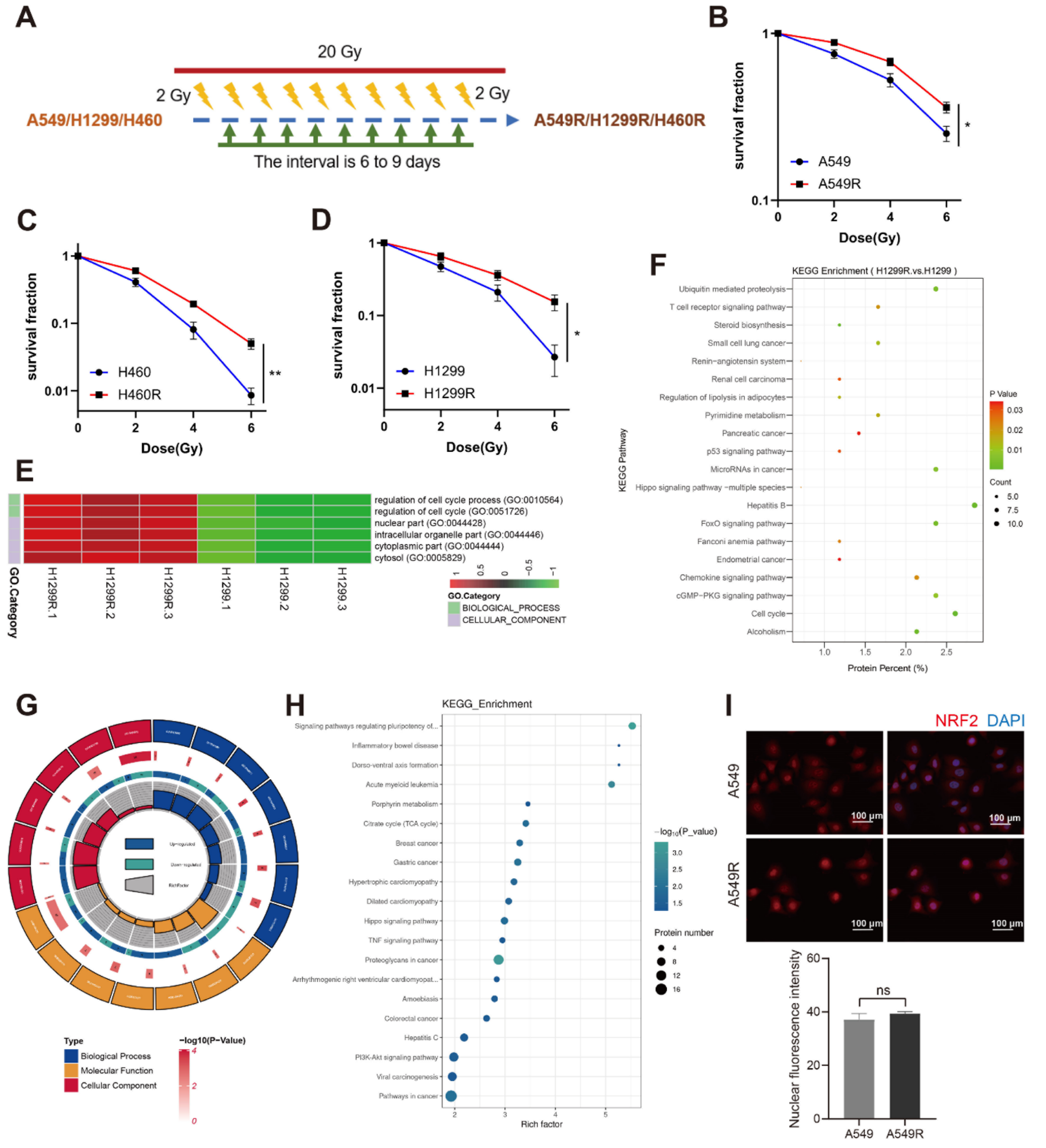

Figure S2. Construction of the radiation-resistant cells.

A Radioresistant cells (A549R/H460R/H1299R) were constructed by the fractionated doses of 2 Gy ^{137}Cs γ-rays (total 20 Gy), with a 6 to 9 days interval between each irradiation. **B-D**

Colony formation efficiency of radioresistant cells (A549R/H460R/H1299R) treated with indicated doses of ^{137}Cs γ-rays. The data are presented as the means ± S.E.M. $^{*}P < 0.05$, $^{**}P < 0.01$. **E** GO heatmap displaying differentially expressed proteins in biological processes and cellular components in H1299 and H1299R cells. **F** KEGG pathway analysis of significantly enriched signaling pathways of differentially expressed proteins in H1299 and H1299R cells. **G** GO analysis of differentially expressed proteins in biological processes and cellular components in H460 and H460R cells. **H** KEGG pathway analysis of significantly enriched signaling pathways of differentially expressed proteins in H460 and H460R cells. **I** Immunofluorescence analysis of NRF2 (red) protein level and nuclear localization changes in A549/A549R cells. Nuclei were visualized by DAPI staining (blue). Nuclear fluorescence intensity was analyzed using ImageJ software. Data are means ± S.E.M. (n = 3). Scale bar: 100 μm.

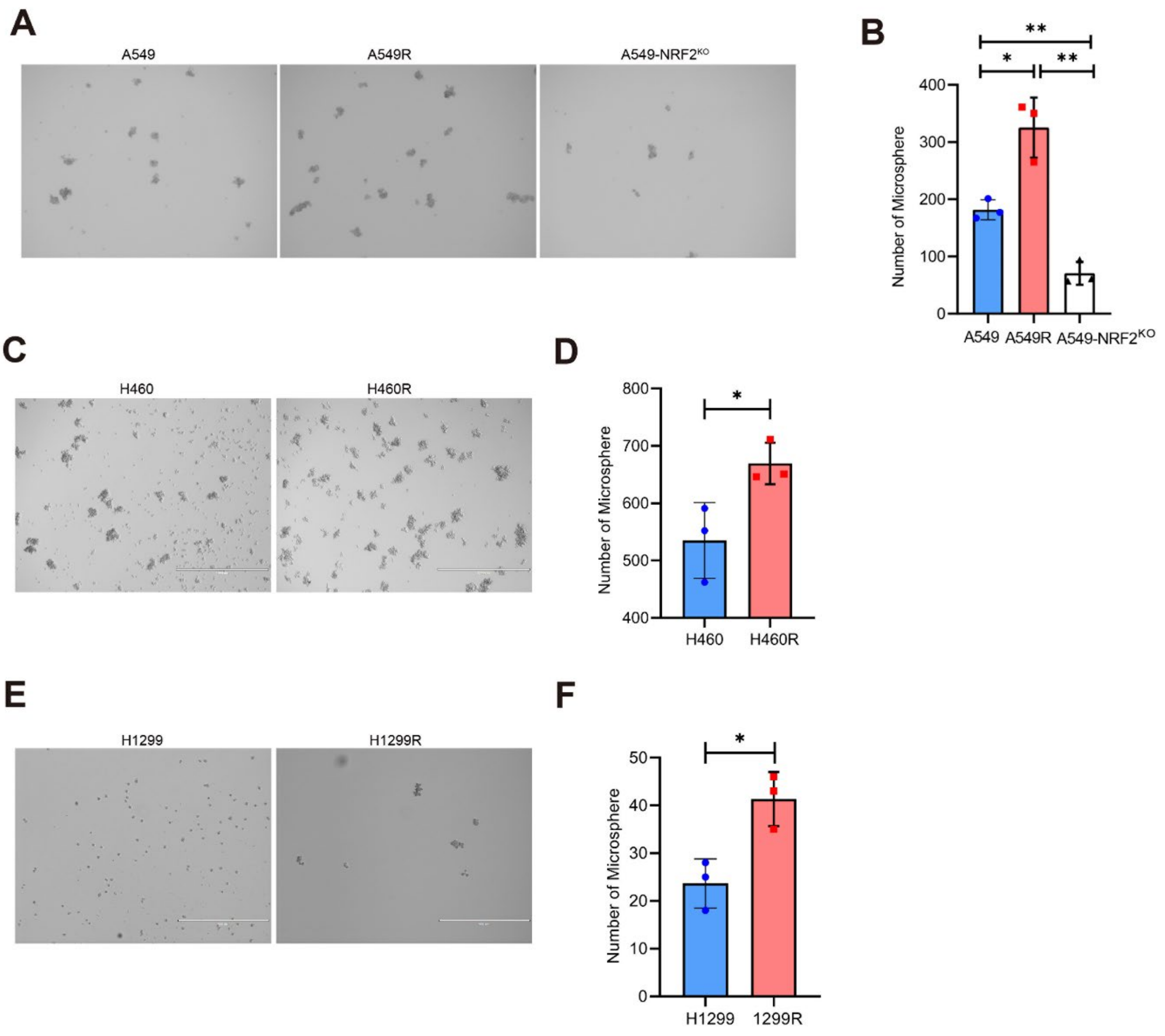

Figure S3. Radiation resistant cells have greater tumor stemness than normal cells.

A, B Comparison of tumor stemness of A549, A549R and A549-NRF2^{KO} cells by tumor stem cell sphere forming assay. Data are means ± S.E.M. (n = 3). *$P < 0.05$, **$P < 0.01$. Scale bar: 1000 μm. **C, D** Comparison of tumor stemness of H460 and H460R cells by tumor stem cell sphere forming assay. Data are means ± S.E.M. (n = 3). *$P < 0.05$. Scale bar: 1000 μm. **E, F** Comparison of tumor stemness of H1299 and H1299R cells by tumor stem cell sphere forming assay. Data are means ± S.E.M. (n = 3). *$P < 0.05$. Scale bar: 1000 μm.

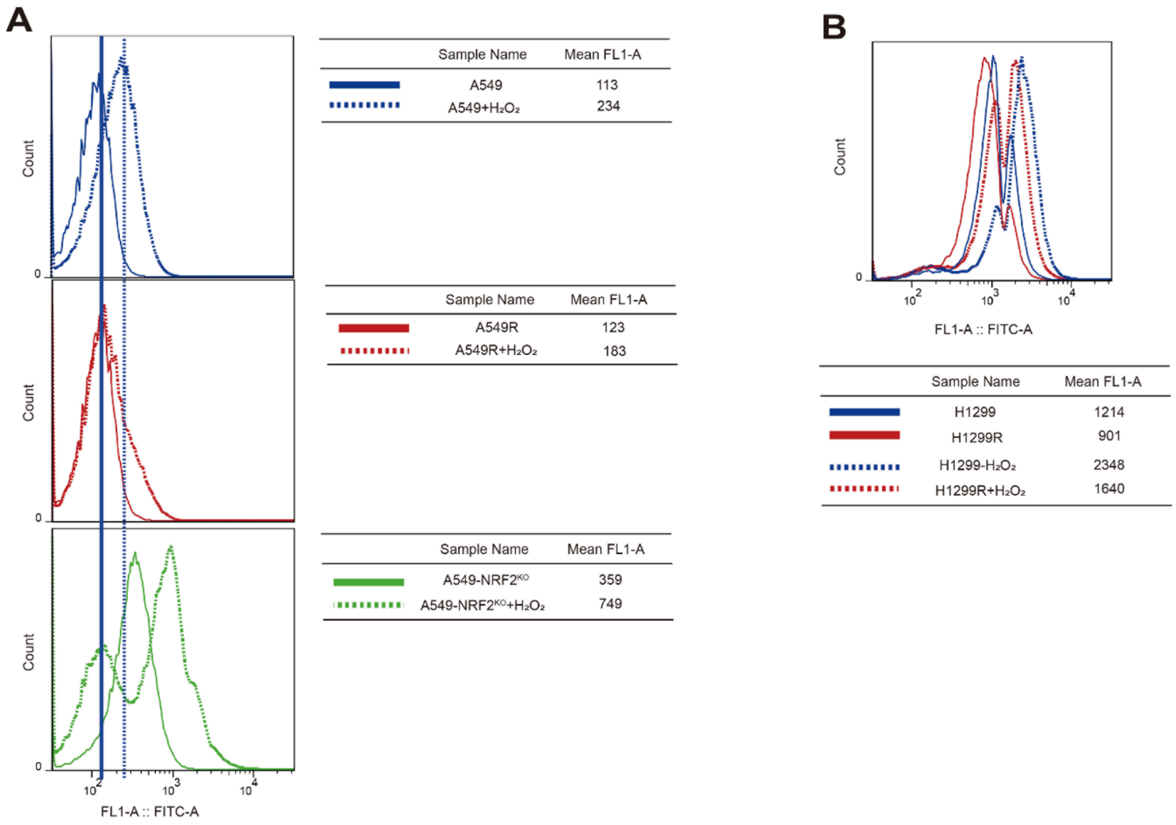

Figure S4. Radiation resistant cells have greater antioxidant capacity than normal cells.

A A549, A549R and A549-NRF2^{KO} cells were pretreated with 500 µM H_2O_2 for 4 h or not. A flow cytometry-based kit was used to detect the level of ROS in cells. **B** H1299 and H1299R cells were pretreated with 500 µM H_2O_2 for 4 h or not. A flow cytometry-based kit was used to detect the level of ROS in cells.

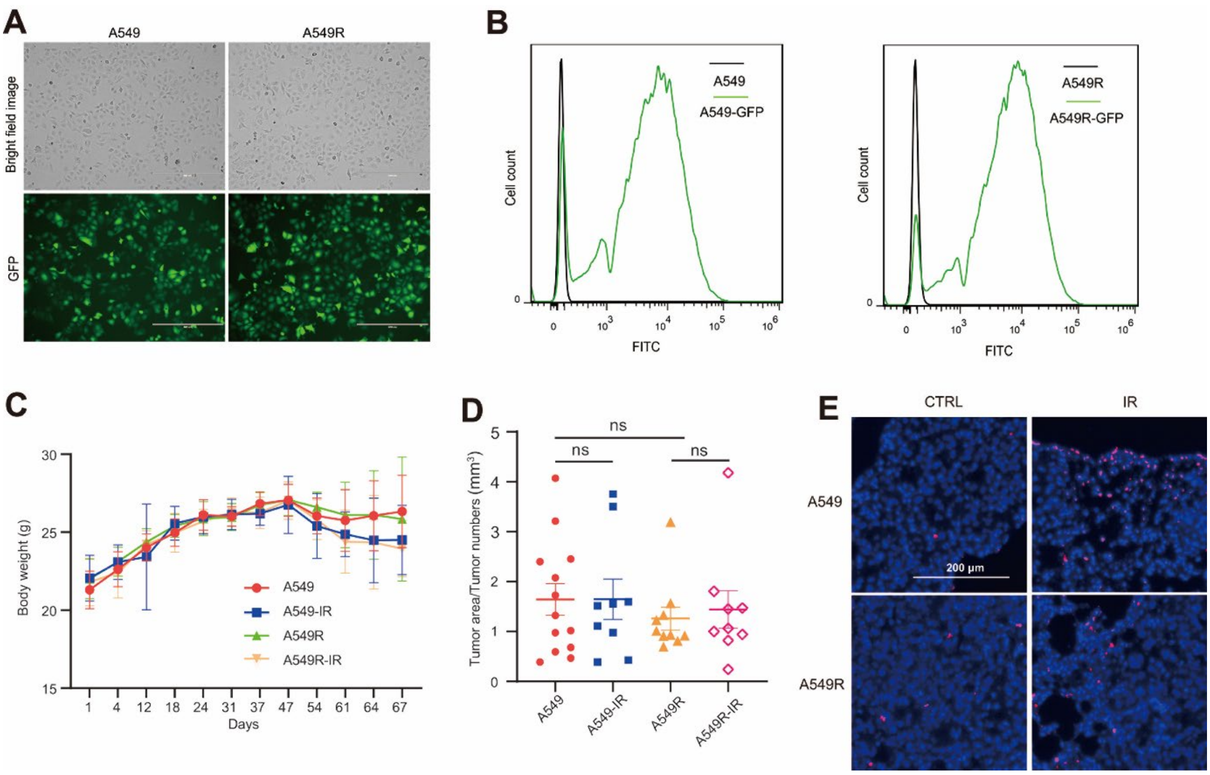

Figure S5. Construction of A549/A549R cells with GFP fluorescence.

A Representative image show the infection efficiency of lentiviruses with GFP expression. Scale bar: 400 μm. **B** Detection of GFP fluorescence intensity of A549 and A549R cells by flow cytometry. **C** Body weight monitoring of nude mice during animal experiments. **D** Statistics of the average area of each tumor per animal (mm^3). The area is calculated using Image J software. The data are presented as means ± S.E.M. ($n > 3$). **E** TUNEL of apoptotic cells in A549/A549R lung metastatic tumor exposed to ^{137}Cs γ-rays.

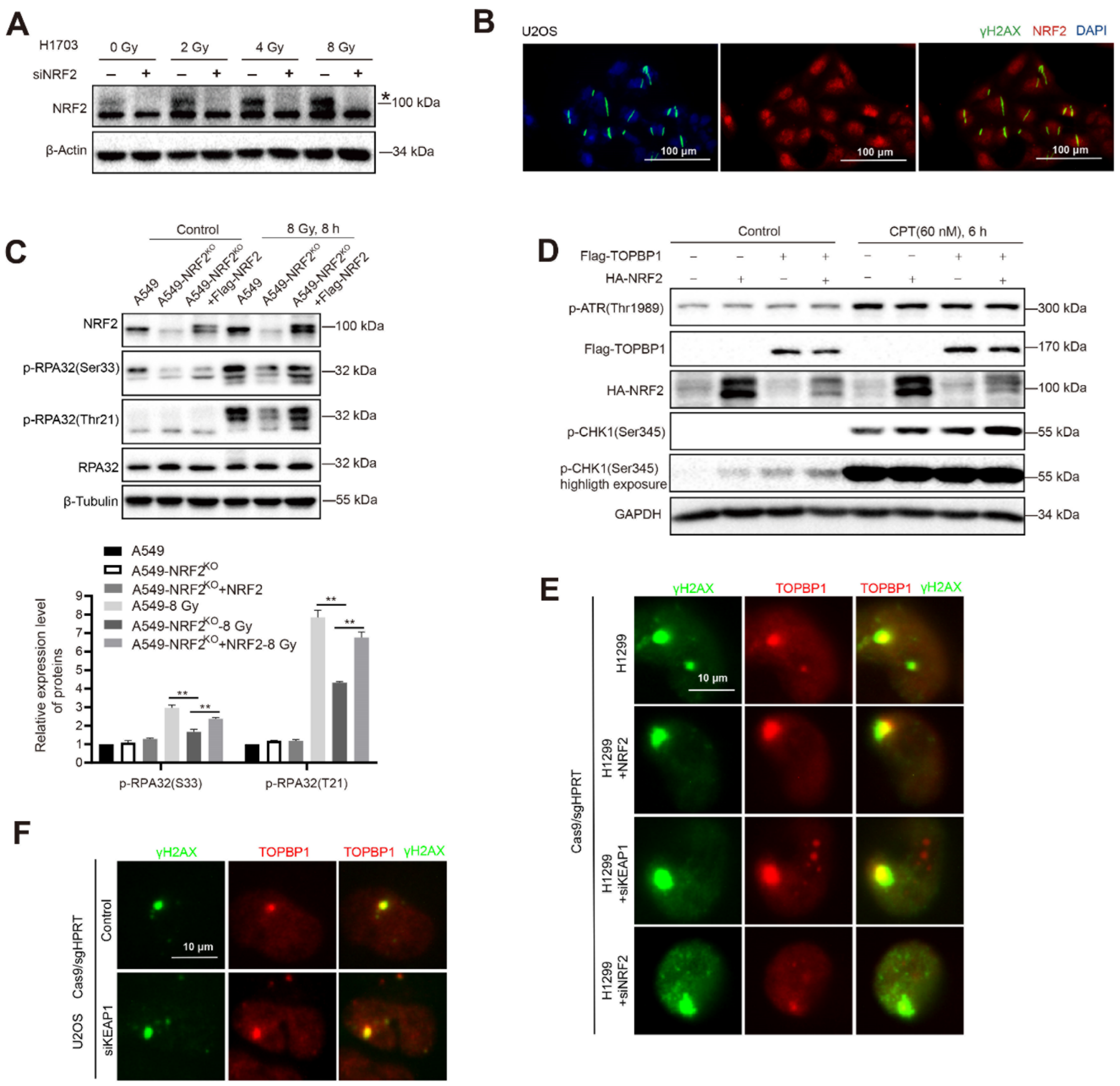

Figure S6. NRF2 affects TOPBP1 recruitment at DNA damage sites after irradiation.

A Western blotting of NRF2 in H1703 cells treated with different doses of ^{137}Cs γ-rays for 8 h. **B** Immunofluorescence analysis of NRF2 (red) in the site of DNA damage (γ-H2AX: green) of U2OS cells caused by laser micro-irradiation. The nuclei were visualized using DAPI staining (blue). Scale bar: 100 μm. **C** Western blotting of NRF2, p-RPA32, and RPA32 in A549, A549-NRF2^{KO}, and A549-NRF2^{KO} + Flag-NRF2 cells treated with 8 Gy ^{137}Cs γ-rays for 8 h. The data are presented as the means ± S.E.M. $^{**}P < 0.01$. **D** Western blotting of p-ATR, TOPBP1, NRF2, p-CHK1 in H1299 cells expressed Flag-TOPBP1 or HA-NRF2, and then treated with

60 nM CPT for 6 h. **E** Immunofluorescence analysis of TOPBP1 (red) in the DNA damage site (γ-H2AX: green) caused by transfected with Cas/sgHPRT in H1299 cells transfected with siNRF2, siKEAP1 and HA-NRF2. **F** Immunofluorescence analysis of TOPBP1 (red) in the DNA damage site (γ-H2AX: green) caused by transfected with Cas/sgHPRT in U2OS cells transfected with siKEAP1.

Table S1. The data from 29 studies of 12,478 samples/9,930 patients with lung cancer were used for gene variant analysis and prognostic survival analysis of NRF2 and KEAP1.

Data sources	Sample numbers
Lung Adenocarcinoma (MSK Mind, Nature Cancer 2022)	247 samples
Lung Cancer (SMC, Cancer Research 2016)	22 samples
Thoracic Cancer (MSK, Nat Commun 2021)	69 samples
Thoracic PDX (MSK, Provisional)	318 samples
Small Cell Lung Cancer (CLCGP, Nat Genet 2012)	29 samples
Small Cell Lung Cancer (Johns Hopkins, Nat Genet 2012)	80 samples
Small Cell Lung Cancer (U Cologne, Nature 2015)	120 samples
Small-Cell Lung Cancer (Multi-Institute, Cancer Cell 2017)	20 samples
Metastatic Non-Small Cell Lung Cancer (MSK, Nature Medicine 2022)	2621 samples
Non-Small Cell Lung Cancer (MSK, Cancer Cell 2018)	75 samples
Non-Small Cell Lung Cancer (MSK, J Cin Oncol 2018)	240 samples
Non-Small Cell Lung Cancer (MSK, Science 2015)	16 samples

Non-Small Cell Lung Cancer (TRACERx, NEJM & Nature 2017)	447 samples
Non-Small Cell Lung Cancer (University of Turin, Lung Cancer 2017)	41 samples
Pan-Lung Cancer (TCGA, Nat Genet 2016)	1144 samples
Lung Adenocarcinoma (Broad, Cell 2012)	183 samples
Lung Adenocarcinoma (CPTAC, Cell 2020)	110 samples
Lung Adenocarcinoma (MSK, 2021)	186 samples
Lung Adenocarcinoma (MSK, J Thorac Oncol 2020)	604 samples
Lung Adenocarcinoma (MSK, NPJ Precision Oncology 2021)	426 samples
Lung Adenocarcinoma (MSK, Science 2015)	35 samples
Lung Adenocarcinoma (OncoSG, Nat Genet 2020)	305 samples
Lung Adenocarcinoma (TCGA, Firehose Legacy)	586 samples
Lung Adenocarcinoma (TSP, Nature 2008)	163 samples
Lung Adenocarcinoma Met Organotropism (MSK, Cancer cell 2023)	2653 samples
Lung Cancer in Never Smokers (NCl, Nature Genetics 2021)	232 samples
Non-Small Cell Cancer (MSK, Cancer Discov 2017)	915 samples
Lung Squamous Cell Carcinoma (CPTAC, Cell 2021)	80 samples
Lung Squamous Cell Carcinoma (TCGA, Firehose Legacy)	511 samples

Table S2. Oligonucleotides used for siRNA.

**Gene**	**Sense (5'-3')**

TOPBP1-siRNA	5′-ACAAAUACAUGGCUGGUUA-3′
ETAA1-siRNA	5′-UGACAAAGCAGUUAGGUAA-3′
RPA70-siRNA	5′-GGAAUUAUGUCGUAAGUCA-3′
RPA32-siRNA	5′-AACCUAGUUUCACAAUCUGUU-3′
NRF2-siRNA	5′-CCGGCATTTCACTAAACACAA-3′
KEAP1-siRNA	5′-ACAACAGUGUGGAGAGGUA-3′

Table S3. Antibodies and dilution ratio.

Antibody	Brand	Catalog No.	Dilution rate
NRF2	Proteintech	16396-AP	1: 2000
ATR	GeneTex	GTX70109	1: 2000
ATR (Thr1989)	GeneTex	GTX128145	1: 3000
ATR (Thr428)	Cell Signaling Technology	#2853	1: 1000
CHK1	GeneTex	GTX100070	1: 5000
CHK1 (Ser317)	Cell Signaling Technology	#12302	1: 1000
CHK1 (Ser345)	Cell Signaling Technology	#2341	1: 1000
KEAP1	Proteintech	10503-2-AP	1: 2000
γ-H2AX (Ser139)	Abcam	ab26350	1: 1000
Heme Oxygenase 1	Abcam	ab137749	1: 2000
NQO1	Abcam	ab28947	1: 1000
53BP1	Abcam	ab36823	1: 10000
BRCA1	GeneTex	GTX70111	1: 2000

ETAA1	Abcam	ab197017	1: 2000
TOPBP1	ThermoFisher	A300-111A-M	1: 5000
ATRIP	Cell Signaling Technology	#2737	1: 1000
RPA32	Abcam	ab101216	1: 2000
RPA32 (Ser33)	Abcam	ab211877	1: 2000
RPA32 (Thr21)	Abcam	ab109394	1: 5000
RPA32 (S4/S8)	Abcam	ab87277	1: 2000
LaminB1	Proteintech	66095-1-Ig	1: 5000
GAPDH	Proteintech	60004-1-Ig	1: 5000
Histone H3	Abcam	ab18521	1: 1000
β-tubulin	Proteintech	66240-1-Ig	1: 5000
FITC-Anti-Mouse IgG (H+L)	Proteintech	SA00003-1	1: 400
FITC-Anti-Rabbit IgG (H+L)	Proteintech	SA00003-2	1: 400
Cy3-Anti-Mouse IgG (H+L)	Proteintech	SA00009-1	1: 400
Cy3-Anti-Rabbit IgG (H+L)	Proteintech	SA00009-2	1: 400
IgG Control	Proteintech	30000–0-AP	1: 1000
HRP-Anti-Mouse IgG (H+L)	Proteintech	SA00001-1	1: 5000
HRP-Anti-Mouse IgG (H+L)	Proteintech	SA00001-2	1: 5000

Table S4. Proteins upregulated simultaneously in H1299R and H460R cells.

**Protein ID**	**Gene**
O60493	SNX3

P04083	ANXA1
P04183	TK1
P04632	CAPNS1
P15559	NQO1
P28482	MAPK1
P40763	STAT3
P50402	EMD
P62993	GRB2
Q14318	FKBP8
Q99497	PARK7
Q9UHD9	UBQLN2
Q9UJU6	DBNL
